# Dual-stage optimizer for systematic overestimation adjustment applied to multi-objective genetic algorithms for biomarker selection

**DOI:** 10.1093/bib/bbae674

**Published:** 2024-12-30

**Authors:** Luca Cattelani, Vittorio Fortino

**Affiliations:** School of Medicine, Institute of Biomedicine, University of Eastern Finland, Yliopistonranta 1, PO Box 1627, 70211 Kuopio, Finland; School of Medicine, Institute of Biomedicine, University of Eastern Finland, Yliopistonranta 1, PO Box 1627, 70211 Kuopio, Finland

**Keywords:** model selection, overestimation adjustment, multi-objective, biomarker discovery, omics, genetic algorithms

## Abstract

The selection of biomarker panels in omics data, challenged by numerous molecular features and limited samples, often requires the use of machine learning methods paired with wrapper feature selection techniques, like genetic algorithms. They test various feature sets—potential biomarker solutions—to fine-tune a machine learning model’s performance for supervised tasks, such as classifying cancer subtypes. This optimization process is undertaken using validation sets to evaluate and identify the most effective feature combinations. Evaluations have performance estimation error, measurable as discrepancy between validation and test set performance, and when the selection involves many models the best ones are almost certainly overestimated. This issue is also relevant in a multi-objective feature selection process where various characteristics of the biomarker panels are optimized, such as predictive performances and feature set size. Methods have been proposed to reduce the overestimation after a model has already been selected in single-objective problems, but no algorithm existed capable of reducing the overestimation during the optimization, improving model selection, or applied in the more general multi-objective domain. We propose Dual-stage Optimizer for Systematic overestimation Adjustment in Multi-Objective problems (DOSA-MO), a novel multi-objective optimization wrapper algorithm that learns how the original estimation, its variance, and the feature set size of the solutions predict the overestimation. DOSA-MO adjusts the expectation of the performance during the optimization, improving the composition of the solution set. We verify that DOSA-MO improves the performance of a state-of-the-art genetic algorithm on left-out or external sample sets, when predicting cancer subtypes and/or patient overall survival, using three transcriptomics datasets for kidney and breast cancer.

## Introduction

Molecular biomarker discovery with machine learning (ML) is usually limited by data that include many features but few samples [1]. This renders trained models prone to overfitting and the evaluation prone to estimation error. Hyperparameter tuning, including feature selection, is often a crucial aspect of the optimization process where the model’s performance is assessed using a training, validation, and test paradigm. This involves choosing the most effective hyperparameters for the ML’s model. Selecting the best model from many often leads to overestimation, given by a significant gap between validation set performance and actual, real-world performance, a phenomenon known as the “winner’s curse”. The models that fit the noise present in the validation set are advantaged, a phenomena sometimes referred as overfitting on the validation set [2]. Seeking higher accuracies by expanding hyperparameter configurations can enhance model performance on validation sets. However, this often results in heightened overestimation, leading to reduced or potentially negative impact on test set performance. For this reason brute force approaches, based on increasing the number of the hyperparameter configurations that are evaluated, often do not provide the desired improvement: the more evaluations are performed, the more overestimation must be expected on the selected ones. In biomarker discovery, the focus is often not only on optimizing the accuracy of ML models, but also on minimizing the number of used molecular features, to ensure clinical feasibility and resource efficiency [3–6]. Characterizing all the best compromises between predictive value and feature set size is a multi-objective (MO) optimization problem [7, 8] that can be solved by means of MO feature selection (MOFS) techniques [4, 6, 9–11]. They aim to identify not just a single best solution, as in single-objective (SO) problems, but rather a Pareto front of solutions: the set of optimal solutions that illustrate the trade-offs between different objectives. Still, all candidate solutions are evaluated on the validation set, which can result in the overestimation of the performance of the selected models.

**Figure 1:**
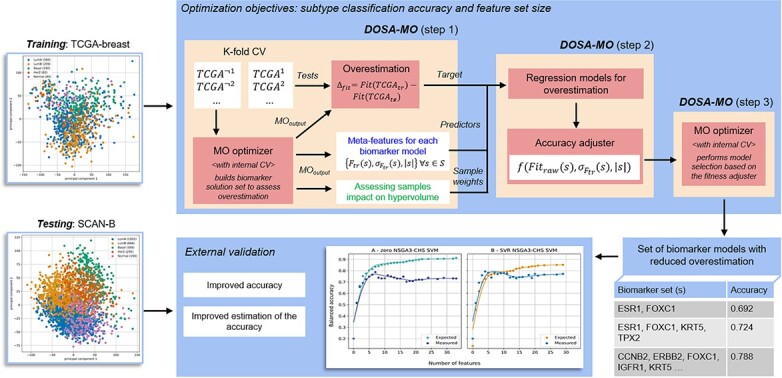
Depiction of a DOSA-MO use case for external validation of breast cancer biomarkers, including its architecture in three steps. A MO problem is defined with two objectives: cancer subtype classification and parsimony in the feature set size. TCGA breast omics data are fed to DOSA-MO for its optimization process. In step 1, it performs a k-fold CV with a wrapped MO optimizer and collects the solutions from all the folds. From each solution and objective a sample is constructed. It has the fitness expected by the wrapped MO, its SD, and the feature set size as independent variables, the overestimation (expected fitness minus fitness assessed on left-out set) as dependent variable, and the partial derivative on the HV with respect to this fitness measurement as sample weight. In step 2, these samples are used to train regression models for overestimation, and new adjusted objective functions are created. In step 3, a wrapped MO optimizer is run with the adjusted objective functions, impacting how the models are selected. DOSA-MO outputs a set of biomarkers, each one with its own set of genes and expected accuracy. The SCAN-B dataset is used to externally validate the set of solutions. Both the accuracy and its estimation improve with respect to the biomarkers identified by the wrapped MO algorithm alone (Section Results).

K-fold cross-validation (CV) stands as the predominant methodology for ML assessment, with its advantages and limitations extensively explored, particularly in SO scenarios [12–14]. K-fold CV returns a model trained on all the available samples and an estimation of its performance computed by averaging $k$ CV results. The obtained model performance tends to be overestimated when multiple configurations are evaluated, and the model (or feature set) with the best performance is returned [2]. This situation becomes particularly pronounced in MO approaches, where identifying multiple favorable trade-offs often leads to an increased number of models to be evaluated. A practical, illustrative example is presented in Cattelani et al. Fig. 1, where the performance of various gene expression-based molecular feature sets for classifying breast cancer subtypes is evaluated [6]. Balanced accuracy and feature set size serve as evaluation metrics. The expected balanced accuracy tends to increase with the addition of more features. Similarly, overestimation—quantified as the difference between expected and test set performance—also grows as the estimation improves. These correlations may suggest that characteristics of the solutions might be predictive of the degree of overestimation, and instead of focusing on just extensively exploring the solution space, which tends to increase the overestimation of the selected solutions, it can be worth to try to reduce their overestimation by predicting it with an ML approach.

Although various methods have been developed to enhance performance estimation in model selection using k-fold CV, their design and implementation have been limited to SO problems. Tsamardinos et al. [15] compared double CV, the Tibshirani and Tibshirani method [16], and nested CV in their ability to improve the estimation of the fitness for SO problems. These algorithms modify fitness estimations but do not affect the model selection process: the chosen model remains the same as it would be using simple hyperparameter optimization with k-fold CV for model evaluation. Automated ML (AutoML) tools offer an approach designed to explore various model and hyperparameter combinations. They aim to identify the most effective model along with an assessment of its performance. In Tsamardinos et al., six AutoML tools were compared [17]. Of these, only one had a predictive performance estimation strategy that could adjust for multiple model validations (limitedly to SO problems and not affecting model selection), while most of the tools have the necessity to withhold a test set for an unbiased estimation of the performance of the winning model, thus losing samples from the final model training.

No significant efforts have been directed toward improving performance estimation in MO problems, of which SO problems are a special case and which are more relevant to biomarker discovery in high-dimensional omics data. Moreover, while there are approaches for SO problems to enhance the performance estimation of a chosen model, these models are still selected based on unadjusted estimations, not leading to any improvement in the actual model selection process. To the best of our knowledge, no previous work experimented the effectiveness of methods for mitigating the overestimation in MO problems using ML algorithms. Additionally, no previous work applied the adjustment to the performance estimation during the optimization of the solution set, thus affecting the selection.

Here we present the DOSA-MO (Dual-stage Optimizer for Systematic overestimation Adjustment in Multi-Objective problems), an algorithm aimed at predicting and adjusting for overestimation in MO problems. Initially, a wrapped MO optimizer is integrated with ML algorithms, to create a series of preliminary solutions (feature sets in our case study). These initial solutions are then used to train regression models that are designed to predict performance overestimation. This prediction is based on characteristics of the solutions, such as the variance in evaluation metrics or the size of the feature set itself. As wrapped MO optimizer for our case study we used a modification of the Non-dominated Sorting Genetic Algorithm III (NSGA3) [18]: NSGA3 with Clone-Handling method and Specialized mutation operator (NSGA3-CHS, see Section 2 Case study overview). Subsequently, the algorithm executes a wrapped MO optimizer once more, but now utilizes the regression models to deliver adjusted fitness evaluations, enhancing the model selection process. The final resulting models are then trained on all the available samples, thus no samples are lost in order to compute their expected performance on new data. Figure 1 shows the steps of the DOSA-MO algorithm in a practical use case. In our benchmarking study, which concentrates on selecting gene expression-based feature sets for cancer subtype classification [10, 19, 20] and patient survival prediction [21], we have empirically demonstrated that DOSA-MO effectively mitigates overestimation and improves model selection. It consistently delivers improved performance estimations in biomarker discovery across diverse population-based cohort datasets. Additionally, two novel measures are introduced in our study: MO performance error (MOPE) and Pareto delta ($P_{\Delta }$). To the best of our knowledge, these are the first to be designed to evaluate the discrepancy between the performance expected by the algorithm and the actual performance observed on new samples in MO problems. These assessments consider the entirety of the solution set determined by the MO optimizer, providing a comprehensive view of the algorithm’s effectiveness.

Disambiguations for the most technical terms can be found in Supplementary Section 4.

## Methods

### DOSA-MO: algorithm description

The DOSA-MO algorithm wraps a MO optimizer, serving two purposes: improve the estimate of the solutions’ performance, and increase the overall performance of the solution set (set of biomarker models in our case study). In our case study, these optimizers are represented by genetic algorithms (GAs) [22] specifically designed for MO problems and paired with supervised ML algorithms, such as the Gaussian Naïve Bayes (NB) classifier to distinguish cancer subtypes or the Cox Proportional-Hazards Model (Cox) for survival analysis. In more details, DOSA-MO consists of three steps (Supplementary Fig. S1a).

**1. Generating solution sets for overestimation prediction.** It collects solutions to be used as training samples to learn how to adjust the objective functions that are used to evaluate solutions, such as the classification accuracy. This consists of running MO optimizers in a k-fold CV loop. For each fold, a solution set is produced using only training data, and its performance is measured on the left-out samples. Depending on the MO optimizers used, step 1 might be computationally expensive. A strategy to limit its cost when using GAs is described in Supplementary Section 1.3.

**2. Training of regression models for overestimation.** For each objective DOSA-MO trains a regression model on the samples collected during step 1. Each sample contains as independent variables three meta-features of the solutions that are potentially predictive of the overestimation. They are the original fitness (i.e. the fitness used by the optimizer to choose the best solutions, measured using only training data, applying inner k-fold CV in our case study), the standard deviation (SD) of that fitness, and the number of features included in the solution (number of genes forming the biomarker in our case study). Our method for computing the SD of the original fitness combining bootstrap with k-fold CV is described in Supplementary Section 1.4. The dependent variable is the overestimation: the difference between the original fitness and the fitness computed on new data through CV. Solutions cannot be considered as equally important. We might expect solutions that are in crowded areas of the non-dominated front to be selected less often by a decision maker. Consequently, each sample is weighted according to the partial derivative of the HyperVolume (HV, constructed using the original fitnesses) [23] with respect to the considered solution and objective. The weights for each fold and objective are scaled to sum to 1. We minimize the absolute error, when allowed by the specific regression model, since the impact of an error on the HV is approximately linear for small errors.

**3. Generating the solution set using adjusted performance.** A second MO optimizer is deployed to generate a solution set (each solution refers to a feature set in our case study), with objectives that are adjusted by previously trained regression models for overestimation. Each original objective function is replaced by a pipeline that initially computes the function’s result, its SD, and the feature count of the solution. These data are then feed to the corresponding adjuster regression model, which predicts the overestimation. The final adjusted performance is calculated by deducting the predicted overestimation from the original fitness value. The solution set generated by the MO optimizer during this final run represents the output of the whole DOSA-MO.

### Pseudocode formulation of the DOSA-MO algorithm

In order to wrap any MO optimizers, the DOSA-MO must be polymorphic, so we define an abstract class MultiObjectiveOptimizer representing a generic MO optimizer (Pseudocode 1). It has a single method optimize that works with provided objectives and training data. The DOSA-MO is a MultiObjectiveOptimizer itself (Pseudocode 2).


\begin{align*} \begin{array}{l}\hline \textbf{Pseudocode 1}\quad \mathtt{MultiObjectiveOptimizer}\ \text{abstract}\ \text{class}\phantom{\quad}\\ \text{definition}.\\\hline \mathtt{class\ MultiObjectiveOptimizer}\\ \quad\mathtt{method\ optimize(objectives,\ trainingData)}\\\hline \end{array} \end{align*}

\begin{align*} \begin{array}{l}\hline \textbf{Pseudocode 2}\quad \mathtt{DosaMO}\ \text{class}\ \text{definition}.\kern7pc\\ \hline \mathtt{class\ DosaMO}\\ \ \quad\qquad \mathtt{inherits\ MultiObjectiveOptimizer}\\ \\ \quad \mathtt{method\ new(}\\ \qquad\qquad\mathtt{tuningOptimizer,\ adjusterLearner,}\\ \qquad\qquad \mathtt{mainOptimizer):}\\ \quad \mathtt{self.tuningOptimizer = tuningOptimizer}\\ \quad \mathtt{self.adjusterLearner = adjusterLearner}\\ \quad\mathtt{self.mainOptimizer = mainOptimizer}\\ \\ \mathtt{method\ optimize(objectives,\ trainingData):}\\ \quad \mathtt{foldsData =}\\ \quad\quad\quad\quad\mathtt{createFolds(trainingData)}\\ \quad\mathtt{foldHofs = [}\\ \quad\quad\quad\quad\mathtt{self.tuningOptimizer.optimize(}\\ \quad\quad\quad\quad\quad\quad\quad \mathtt{objectives,\ f.train)}\\ \quad\quad\quad\quad \mathtt{for\ f\ in\ foldsData]}\\ \mathtt{for\ i\ in\ 1::objectives.size:}\\ \quad\quad \mathtt{obj = objectives[i]}\\ \quad\quad \mathtt{weights = [}\\ \quad\quad \quad \mathtt{assignWeights(}\\ \quad\quad\quad \mathtt{h.fitnessHyperboxes(),\ i)}\\ \quad\quad\quad \mathtt{for\ h\ in\ foldHofs]}\\ \quad\quad \mathtt{adjuster = trainAdjuster(}\\ \quad\quad\quad\quad\quad\quad \mathtt{self.adjusterLearner,\ obj,}\\ \quad\quad\quad\quad\quad \mathtt{\quad foldHofs,\ foldsData,\ weights)}\\ \quad\quad \mathtt{adjustedObjectives[i] =}\\ \quad\quad\quad\quad\quad\quad \mathtt{createAdjustedObjective(}\\ \quad\quad\quad\quad\quad\quad\quad\quad \mathtt{obj,\ adjuster)}\\ \mathtt{return\ self.mainOptimizer.optimize(}\\ \quad\quad\ \mathtt{adjustedObjectives,\ trainingData)}\\\hline \end{array} \end{align*}

The method new is just a simple constructor that saves the polymorphic parts of the algorithm: the tuningOptimizer, a MO optimizer used to create the samples for the adjusting regression, the actual regression model (adjusterLearner), and the mainOptimizer that is the MO optimizer that uses the adjusted objectives to produce the results for the user.

The DosaMO implementation of optimize first organizes the data into folds. The resulting object, foldsData, contains the data itself and also the description of how it is partitioned into folds. DosaMO then executes the tuningOptimizer on each fold and collects the results: a set of solutions for each fold. The set of solutions returned is optimizer-dependent in general, but in our experiments we used the non-dominated front of all the solutions that were explored. For each objective obj the algorithm assigns weights to the solutions: for each tuningOptimizer result set h, the solutions receive a weight that is proportional to the partial derivative of the HV of the belonging result set with respect to the solution and the current objective obj. An adjuster regression model is trained for the current objective obj using the function trainAdjuster that receives in input a regression model adjusterLearner, the current objective obj, the tuningOptimizer solution set for each fold, the data including folds information (foldsData), and the weigths of the samples (weights). The returned adjuster predicts how much the fitness of a solution changes between the training sample set and an unseen testing sample set. The function trainAdjuster has its own description below. Using the previous objective obj and the adjuster, a new adjusted objective is created that when evaluating a solution first uses the old fitness function to compute a temporary fitness and its SD, then adjustes this fitness by subtracting the prediction of the adjuster. Finally, DosaMO runs the mainOptimizer on the whole trainingData, using the adjusted objectives instead of the original objectives.

The trainAdjuster function trains a fitness adjuster regression model for one of the objectives using as samples the solutions resulting from running the tuningOptimizer on all the folds defined by foldsData. Each solution is assigned a weight proportional to the HV partial derivative with respect to the considered objective, with the weights for each fold that sum to 1.


\begin{align*} \begin{array}{l}\hline \textbf{Pseudocode 3}\quad\mathtt{trainAdjuster}\ \text{function}\ \text{definition}.\\\hline \mathtt{trainAdjuster(}\\ \qquad \qquad\mathtt{adjusterLearner,\ obj,\ foldHofs,}\\ \qquad\qquad \mathtt{foldsData,\ weights):}\\ \qquad\mathtt{for\ i\ in\ 1::foldsData.size:}\\ \qquad\qquad \mathtt{allFitnesses =}\\ \qquad\qquad\qquad\qquad \mathtt{evaluateWithCV(}\\ \qquad\qquad\qquad\qquad\qquad \mathtt{foldHofs[i],\ obj,\ foldsData[i])}\\ \qquad\qquad \mathtt{originalFitnesses[i] = allFitnesses.innerCV())}\\ \qquad\qquad \mathtt{stdDevs[i] = allFitnesses.innerCV\_sd()}\\ \qquad\qquad \mathtt{testFitnesses[i] = allFitnesses.test()}\\ \qquad\qquad \mathtt{nFeatures[i] =}\\ \qquad\qquad\qquad\qquad \mathtt{[h.num\_features()\ for\ h\ in\ foldHofs[i]]}\\ \mathtt{return\ adjusterLearner.fit(}\\ \qquad\qquad \mathtt{originalFitnesses,\ stdDevs,\ nFeatures,}\\ \qquad\qquad \mathtt{testFitnesses,\ weights)}\\\hline \end{array} \end{align*}

For each fold i as defined by foldsData, trainAdjuster prepares the samples for training the adjuster regressor (Pseudocode 3). The samples are prepared separately for each fold, then used together in training. The function evaluateWithCV assigns two fitnesses to each solution: one previously computed on the train data of the current fold i (using a nested k-fold CV in our case study), and another computed on the test data. It also computes the SD of the train fitness with the bootstrap method (Supplementary Section 1.4). The differences between train and test performance are the values that the regression will learn to predict. The regression has three input meta-features: the original fitness, i.e. the performance on the train data, the SD of the original fitness, and the number of the features that are included in the solution (number of genes in our case study).

### Case study overview

We have benchmarked DOSA-MO in the context of MOFS for cancer biomarker discovery. Our goal was to identify biomarkers for classifying cancer subtypes and survival prediction in kidney and breast cancer patients. This was done using gene-based kidney and breast transcriptomic datasets from The Cancer Genome Atlas (TCGA) project [24]. The TCGA breast dataset contains 1081 samples. The TCGA kidney dataset contains 793 samples with both subtype and survival information, which increases to 924 when only subtype information is needed. For breast cancer, an additional cohort from The Sweden Cancerome Analysis Network - Breast (SCAN-B) [25] with 2969 samples serves as an external validation set. A description of the datasets and their preprocessing is in Supplementary Section 1.6. The first two principal components of the considered datasets are shown in Supplementary Fig. S2, S3, S5 and S6, while Supplementary Fig. S4 shows the kidney survival outcomes. As wrapped MO optimizer, we used a novel modification of NSGA3 [18]: NSGA3-CHS, with NB or support vector machine (SVM) as inner classifiers, and Cox as inner survival model. We used both internal k-fold CV and external validation to compare the unadjusted optimizer (abbreviated as “zero”) with adjustments by five different regression models: weighted median (dummy), pruned decision tree (ptree), random forest (RFReg), support vector regression (SVR), and SVR with optimized regularization parameters (rSVR). The regression models are described in detail in Supplementary Section 1.2. Supplementary Fig. S1b depicts the external validation procedure.

NSGA3-CHS is an instantiation of the more general algorithm NSGA*. They are both defined in Supplementary Section 1.1. The considered experimental setups are listed in Table 1 and described in detail in Supplementary Section 1.7, where it is also defined the root-leanness, used as fitness function for the parsimony of the feature sets. Classification and survival prediction performance are measured, respectively, with balanced accuracy and concordance index (c-index).

**Table 1:** Overview of datasets, objectives, inner models, and validation methods used in the analysis of kidney and breast cancer

Datasets	Objectives	Inner models	Validation
Kidney cancer	Subtype classification (balanced accuracy), set size (root-leanness)	NB	CV
Kidney cancer	Overall survival (c-index), set size (root-leanness)	Cox	CV
Kidney cancer	Subtype classification (balanced accuracy), overall survival (c-index), set size (root-leanness)	NB/Cox	CV
Breast cancer	Subtype classification (balanced accuracy), set size (root-leanness)	NB, SVM	CV
Breast cancer	Subtype classification (balanced accuracy), set size (root-leanness)	NB, SVM	External (SCAN-B)
Breast cancer	Overall survival (c-index), set size (root-leanness)	Cox	CV

### Measuring overestimation in multi-objective problems

We propose two new methods to measure the error of the performance estimates for the solutions to MO problems: MOPE and $P_{\Delta }$. The data-driven approach complicates the measurements, as CV yields varied performance metrics. These include the performance anticipated by the optimizer, based on training data, and the performance measured on left-out/new samples post-optimization. To our knowledge, there is no established metric for the error in evaluating the predictive performance of solutions in MO problems within a CV framework. In SO scenarios, one might simply assess the absolute difference between the fitness expected from training data and the fitness observed on new data. However, in MO scenarios, it is crucial to consider each solution’s contribution to the Pareto front. We introduce two novel metrics to measure this estimation error in MO CV setups.

#### Multi-objective performance error

We define the MOPE $E_{\upsilon }$ starting from the HV [23] computed on the train performance ($H_{\iota }$) and the Cross HyperVolume (CHV) ($H_{\upsilon }$). $H_{\iota }$ and $H_{\upsilon }$ have been formally defined by Cattelani et al. [10]. Since $H_{\upsilon }$ is a family of functions, with the specific instantiation that depends on the user provided function $\upsilon $, $E_{\upsilon }$ is a family of functions too.


(1) \begin{align*}& E_{\upsilon}(X,X^{\prime})=\lvert{H_{\iota}(X,X^{\prime})-H_{\upsilon}(X,X^{\prime})\rvert}\end{align*}

where $X$ encodes the performance of the solutions evaluated on the train data, and $X^{\prime}$ the performance evaluated on the test data. In our case study we use the same instantiation of $\upsilon $ as in Cattelani et al.: the function $\lambda $ [10].

$E_{\upsilon }$ has a simple definition and can be computed very efficiently if the experimental setup already includes the measuring of $H_{\iota }$ and $H_{\upsilon }$. It can be seen intuitively as the difference between the aggregated performance of all the solution set as expected by the optimizer taking into account only train data, and the aggregated performance of the same solutions when applied on never before seen test data by decision makers that must choose their preferred solution informed by train performance only.

#### Pareto delta ($P_{\Delta }$)

The MOPE, derived directly from the training HV and CHV, does not specify the exact sources of discrepancies between these measures. Although CHV accounts for differences between training and testing performance, variations in solution performance of opposite sign can offset each other, resulting in a lower MOPE despite significant train-test discrepancies. To tackle this issue, we introduce a supplementary metric, the $P_{\Delta }$. It has the property of being equal to $0$ only when there is no difference in performance between train and test data for all elements of the solution set. To calculate the $P_{\Delta }$, we sum up the absolute error in fitness estimation for each solution, multiplying it by the derivative of the HV concerning that specific objective and solution. Then, we compute the average across all objectives.

Let $n$ be the number of solutions and $m$ the number of objectives. Let $X$ be an $n\times m$ matrix where $x_{i,j}$ is the train performance for the $j$th objective of the $i$th solution, with $1\le i\le n$ and $1\le j\le m$. Similarly, let $X^{\prime}$ be an $n\times m$ matrix where $x^{\prime}_{i,j}$ is the test performance for the $j$th objective of the $i$th solution. Let $H_\iota $ be the HV computed on the train performance $X$. $\partial H_\iota / {\partial x_{i,j}}$ is the partial derivative of the HV with respect to $x_{i,j}$. We define the $P_{\Delta }$ function as


(2) \begin{align*}& P_{\Delta}(X,X^{\prime})=\frac{1}{m} \sum_{j=1}^{m} \sum_{i=1}^{n}\lvert{x_{i,j}-x^{\prime}_{i,j}\rvert}\frac{\partial H_\iota} {\partial x_{i,j}}\end{align*}

in the special cases with $0$ dimensions or $0$ solutions, we define $P_{\Delta }$ as $0$. As long as the differences $\lvert{x_{i,j}-x^{\prime}_{i,j}\rvert }$ are small, $P_{\Delta }$ is proportional to the difference between the volume of the geometrical union of the space under the train and test fronts and the volume of the intersection of the same two spaces.

## Results

**Figure 2:**
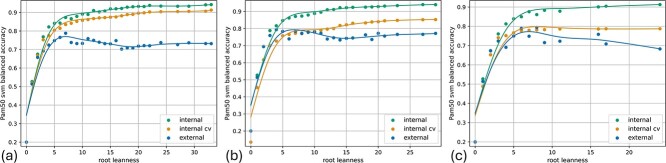
Scatter plots depicting solutions from external validation on breast cancer transcriptomics data using SVM as inner model. MO optimization of balanced accuracy for subtypes prediction and root-leanness. Horizontally, the number of features is depicted for simplicity. For each solution it is shown the performance measured in the inner CV, i.e. the performance expected by the optimizer, the performance of the model trained on the TCGA breast set and tested on the same set, and the performance of the same model on the external SCAN-B set. The lines are interpolating splines. (a) Using the unadjusted optimizer. (b) Using SVR as regression model for fitness adjustment. (c) Using RFReg as regression model for fitness adjustment.

We compare six different instantiations of the DOSA-MO by varying the regression model used for the adjustment, including the zero regression model, equivalent to not applying any adjustment. The regression models receive as input three meta-features of the solutions correlated with the overestimation: the original fitness, its SD, and the feature set size (number of genes). For an example of correlations between meta-features and overestimation see Supplementary Section 2.1 and Supplementary Fig. S8. We use NSGA3-CHS as wrapped model. The tests are repeated with eight different combinations of validation type, datasets, objectives, and classification inner model. For each combination we report two measures for the accuracy of the fitness estimation: the MOPE (Section Comparative analysis using multi-objective performance error) and the $P_{\Delta }$ (Section Comparative analysis using the Pareto delta). Additionally, we report a measure of overall performance of the optimizers: the CHV (Section Evaluating DOSA-MOs effectiveness in MO feature selection). A focus on the best solution sets from the external validation is shown in Fig. 2 and discussed in Supplementary Section 2.2, with in depth performance measures for the classification of each cancer subtype in Supplementary Fig. S9. A more detailed comparison of the quality of the solution sets obtained with and without DOSA-MO is presented in Section Comparison between the best solution sets with and without DOSA-MO, while an analysis of the specific biomarkers that have been identified can be found in Supplementary Section 2.3.

### Overestimation in feature selection for biomarker discovery

We measured the error of the performance estimates with two methods: the MOPE and the $P_{\Delta }$. The MOPE quantifies the discrepancy between the HV computed on the train performance and the CHV. In contrast, the $P_{\Delta }$ captures differences between expected performance and actual performance on new samples, focusing on the more granular level of individual solutions.

#### Comparative analysis using multi-objective performance error

**Figure 3:**
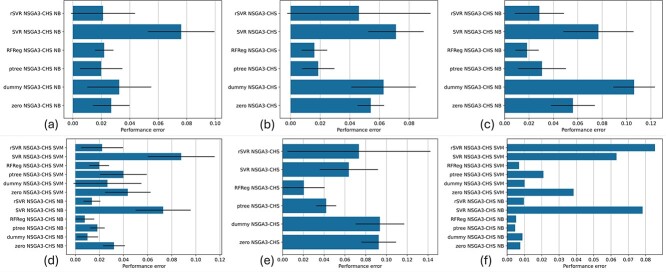
MOPE results for internal k-fold CV (a–e) and external validation (f). (a) Kidney cancer, subtype classification, and root-leanness. (b) Kidney cancer, overall survival prediction, and root-leanness. (c) Kidney cancer, overall survival prediction, subtype classification, and root-leanness. (d) Breast cancer, subtype classification, and root-leanness. (e) Breast cancer, overall survival prediction, and root-leanness. (f) External validation for breast cancer, subtype classification, and root-leanness. Error bars represent SD between folds.

The MOPE evaluates the DOSA-MO algorithm’s capability in reducing the performance estimation error (Section Multi-objective performance error). The MOPE outcomes from various regression-based adjusters are reported in Fig. 3.

Significantly, ptree and RFReg often outperform other overestimation predictors. Therefore, for users prioritizing the reduction of MOPE, ptree and RFReg regressors emerge as particularly effective choices. SVR-based estimators of overfitting appear to be the least effective. ptree and RFReg regressors perform particularly well in external validation scenarios, where an external dataset (SCAN-B) is used to evaluate the models (biomarker sets) that were selected according to their adjusted performance learned from the initial TCGA breast cohort.

#### Comparative analysis using the Pareto delta

**Figure 4:**
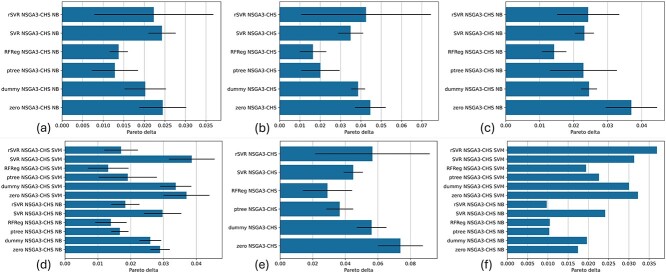
$P_{\Delta }$ results for internal k-fold CV (a–e) and external validation (f). (a) Kidney cancer, subtype classification, and root-leanness. (b) Kidney cancer, overall survival prediction, and root-leanness. (c) Kidney cancer, overall survival prediction, subtype classification, and root-leanness. (d) Breast cancer, subtype classification, and root-leanness. (e) Breast cancer, overall survival prediction, and root-leanness. (f) External validation for breast cancer, subtype classification, and root-leanness. Error bars represent SD between folds.

The $P_{\Delta }$ measure (Section Pareto delta ($P_{\Delta }$)) considers the difference between a model’s predicted and actual test performance for each individual solution. This makes it particularly relevant and more informative than the MOPE in the common situation where users are presented with multiple solutions but will ultimately select only one. The $P_{\Delta }$ across the experimental setups is shown in Fig. 4.

ptree and RFReg regressors consistently outshine others when reducing the $P_{\Delta }$. Interestingly, while no significant differences are noted in MOPE between the dummy model and the unadjusted optimizer, all kidney cancer setups with proper regressors have a lower $P_{\Delta }$ compared to the unadjusted optimizer. rSVR performs better than the zero regressor in all scenarios except one. RFReg and ptree always yield lower $P_{\Delta }$s than the zero regressor, including in breast cancer setups, highlighting their promise in both overestimation measures.

### Evaluating DOSA-MO’s effectiveness in MO feature selection

**Figure 5:**
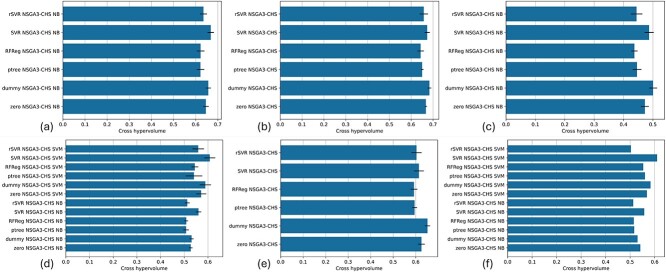
CHV results for internal k-fold CV (a–e) and external validation (f). (a) Kidney cancer, subtype classification, and root-leanness. (b) Kidney cancer, overall survival prediction, and root-leanness. (c) Kidney cancer, overall survival prediction, subtype classification, and root-leanness. (d) Breast cancer, subtype classification, and root-leanness. (e) Breast cancer, overall survival prediction, and root-leanness. (f) External validation for breast cancer, subtype classification, and root-leanness. Error bars represent SD between folds.

The previous results highlight DOSA-MO’s success in reducing performance estimation errors in ML-driven feature selection for biomarker discovery. It is also important to evaluate if DOSA-MO enhances the quality of the produced feature sets: the final output for biomarker selection models considered for clinical validation. To assess the impact on the overall feature selection process, we calculated the CHV for each experimental setup (see Fig. 5).

Tree-based regressors reduce estimation errors but do not significantly improve the quality of solution sets generated by DOSA-MO. This indicates that using these regressors for overestimation prediction may not always enhance solution sets, despite reducing quality estimation errors. Interestingly, dummy and SVR-based MO optimizers consistently outperform the CHV of unadjusted models, highlighting the effectiveness of overestimation prediction for superior feature selection outcomes. Specifically, SVR yelds the best CHV in all setups except in scenarios involving the overall survival objective, while the dummy model excels in setups that include survival prediction.

The HV metric does not take into account the differences between expected and measured performance and is less interesting for ML applications than the CHV [10]. Nonetheless, we present the HVs computed on the test or external sets in Supplementary Section 2.4 and Supplementary Fig. S10.

### Comparison between the best solution sets with and without DOSA-MO

**Figure 6:**
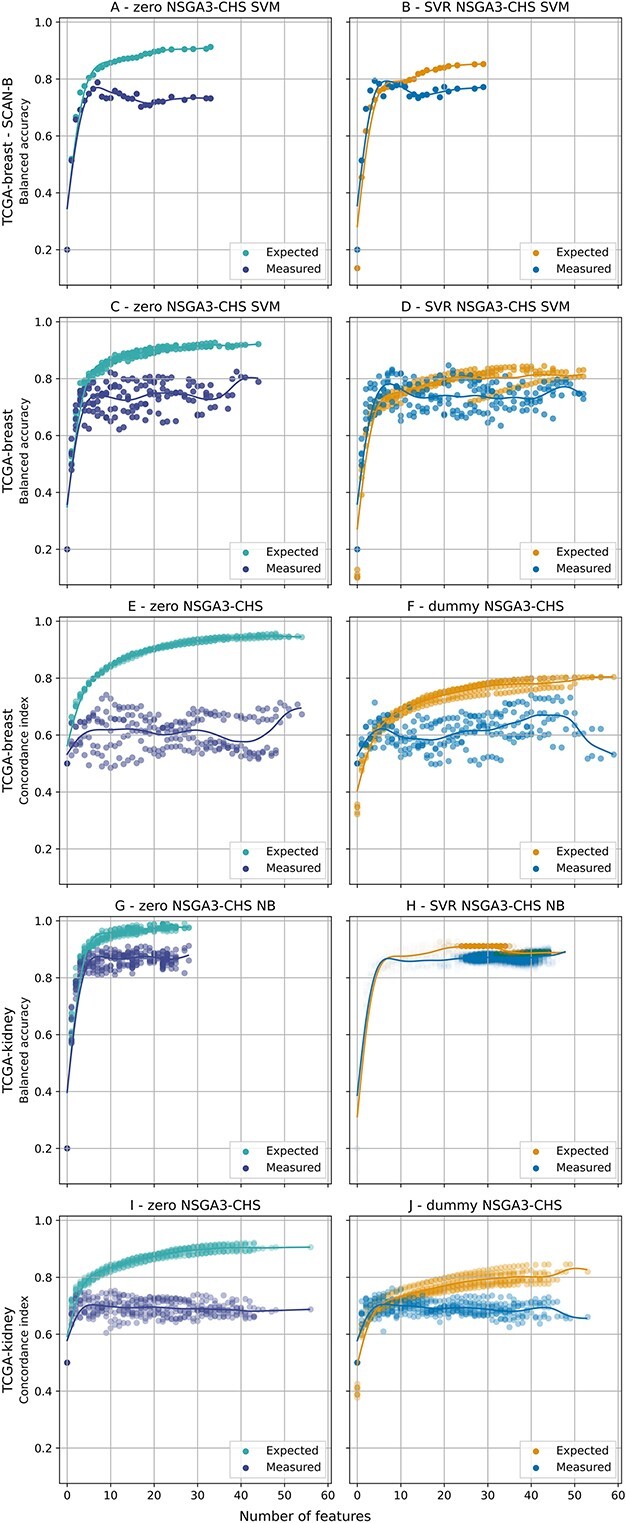
Comparisons between the best solution sets, according to the CHV metric, without using DOSA-MO (on the left) and using DOSA-MO (on the right) for the experimental setups with two objectives. There is a row for each experimental setup. For each result set the subplot shows the performance predicted by the MO optimizer (”Expected”) and the performance measured on left out or external samples (”Measured”). The first row (A, B) shows the external validation with training on TCGA-breast and testing on SCAN-B. The following rows are related to the k-fold CVs on, in turn, TCGA-breast with balanced accuracy and root-leanness objectives (C, D), TCGA-breast with concordance index and root-leanness (E, F), TCGA-kidney with balanced accuracy and root-leanness (G, H), and TCGA-kidney with concordance index and root-leanness (I, J). The subplots for the k-fold CV show the solutions for all the folds. Above each subplot it is indicated the name of the algorithm that produced the represented solution set/sets.

Figure 6 shows, for each experimental setup with two objectives, a comparison between the best solution sets, according to the CHV metric, obtained without DOSA-MO and the ones obtained using DOSA-MO. The non-adjusted algorithms solution sets, on the left, in which the expected fitness is computed by inner k-fold CV, are affected by an overestimation (difference between expected and measured fitness) that increases with the number of features and reaches important amplitudes varying from ${\sim }0.1$ to ${\sim }0.3$ depending on the experimental setup. It can be appreciated that the algorithms with DOSA-MO, on the right, in which the expected fitness is computed by inner k-fold CV followed by fitness adjustment, have a noticeably lower overestimation, thus offering more precise expectations to the user.

The improvement in the overall performance of solution sets obtained by using DOSA-MO, as measured by the CHV (Fig. 3), is more pronounced in the experimental setups for breast cancer subtype classification, followed by the one for breast cancer survival prediction, while the improvement still exists, but is smaller, in the kidney experiments. Coherently, in Fig. 6, the differences in measured fitness between the solution sets obtained without DOSA-MO (A, C) and with DOSA-MO (B, D) for the subtype classification on breast datasets are particularly noticeable, followed by the differences that can be observed between the survival predictions for the same cancer type (E, F). The improvements that are present in the kidney experiments are the less noticeable (G, H, I, J).

## Discussion

We introduced DOSA-MO, a novel wrapper MO optimizer designed to adjust performance measures for overestimation in ML-driven MO problems, improving the solution sets that are produced. This approach has been successfully validated across multiple transcriptomic-based datasets commonly used for biomarker discovery in cancer subtype classification and survival prediction. The assessment has been made in a fully transparent and repeatable manner by providing access to a public repository that includes a working implementation of DOSA-MO as a software tool, the source code, the input data and the results for all the tests that are described in this work. Our benchmarking extends to a scenario where biomarker models, trained in one population-based cohort (TCGA), are applied to classify cancer subtype in a second cohort (SCAN-B). This method effectively enables biomarker discovery and validation across multiple cohorts. External validation is the strongest form of ML-based validation, and in this case, it is particularly robust due to the large sample sizes of the TCGA and SCAN-B cohorts, comprising 1081 and 2969 patients, respectively. Additionally, we have developed two innovative measures for evaluating the performance of MO algorithms: the MOPE and the $P_{\Delta }$. According to both metrics, the DOSA-MO algorithm demonstrated improved performance estimates in all tested cases, particularly when using decision tree-based regression models for predicting overestimation.

Our study found that even a basic regression model, which learns solely the weighted average of overestimation and is used for fitness adjustment, resulted in improved overall performance compared to the unadjusted optimizer in seven out of eight experimental setups, as indicated by the CHV metric. Likewise, the MO optimizer directed by the SVR model, which predicts overestimations, surpassed the unadjusted optimizer in seven out of eight setups, affirming its effectiveness. Notably, the MO optimizer guided by the simpler dummy regression model excelled in the three setups involving survival prediction, while the SVR model proved superior in the five setups focused on subtype classification and feature set size. The rSVR regression model, which selects the hyperparameters for the regularization strength of the SVR with random search, utilized for predicting overestimation, underperformed with respect to dummy and SVR regression models. A regularization that works well while cross-validating on solutions obtained by an unadjusted optimizer is not as effective when the GA runs with a bigger population, more generations, and the adjustment is applied to the evaluation of the individuals.

Predicting the overestimation is more complex when the adjustment to the fitness is applied during the optimization as in our approach. The fitness adjustments alter the optimizer’s exploration path, leading to a divergence in the solution distribution from the one used to train the regressors for overestimations, even within their applicability domain. This creates a “moving target” problem, commonly addressed in other contexts, like artificial neural networks, through incremental optimization across multiple epochs. Although executing more computationally intensive MO GAs is possible, further research is essential to enhance our understanding and address the challenges of the applicability domain and the moving target problem in these contexts.

DOSA-MO is versatile and can be applied to any ML problem, including the ones requiring a MO evaluation framework, making it particularly well-suited for biomarker discovery in high dimensional molecular datasets. It has been experimentally validated with cancer transcriptomics data, demonstrating its applicability and effectiveness to push forward the state of the art in this important domain. Further research will be needed to assess the efficacy of DOSA-MO for other cancer types, wrapping other MO algorithms, with different or multiple data types, like single cell RNA-seq or multiomics, and on other problem domains.

**Key Points**

- We introduce DOSA-MO, the first data-driven, multi-objective optimization algorithm designed to minimize the performance overestimation of the best models. By directly addressing overestimation during the optimization process, our approach ensures more accurate and reliable model selection.
- DOSA-MO identifies solutions with better predictive performance, but it also provides a more accurate estimate of them, allowing the user to have both better solutions and more realistic expectations.
- We verify that DOSA-MO is effective in feature selection for omics-based biomarker discovery, improving the performance on left-out or external sample sets, when predicting cancer subtypes and/or patient overall survival, using gene expression-based datasets for kidney and breast cancer.
- Focusing on complex prediction tasks, such as cancer subtype classification and survival prediction with gene expression data, DOSA-MO uncovers biomarker panels with better predictive ability on new data, marking a crucial step toward advancing these panels closer to the clinical validation stage.

## Supplementary Material

supplementary_bbae674_new

## Data Availability

The original gene expression data used in this study are from public repositories. The preprocessed data, source code, and detailed numerical results are available in a public server (https://github.com/UEFBiomedicalInformaticsLab/BIODAI/tree/main/DOSA_MO, see Supplementary Section 3).
