# Supplementary material for: Dual-stage optimizer for systematic overestimation adjustment applied to multi-objective genetic algorithms for biomarker selection
Source: Brief Bioinform. 2024 Dec 30;26(1):bbae674. doi: 10.1093/bib/bbae674 (PMC11684899; doi:10.1093/bib/bbae674)
Supplement: supplementary_bbae674_new [file supplementary_bbae674_new.pdf]

Dual-stage optimizer for systematic overestimation adjustment applied to multi-objective genetic algorithms for biomarker selection, supplementary material

Luca Cattelani*[1] and Vittorio Fortino†[1]

[1]Institute of Biomedicine, School of Medicine, University of Eastern Finland, Kuopio, 70211, Finland.

1 Supplementary methods

1.1 NSGA* algorithm

The DOSA-MO is a wrapper algorithm that uses an MO optimizer to produce the training data for the regression models and a (possibly different) MO optimizer that will run with the adjusted objectives to produce the final results for the user. In our case study we used NSGA3-CHS algorithm as both tuning and main optimizer. The parameters of population size and number of generations were lower for the tuning optimizer in order to reduce the computational load. Modified Non-Dominated Sorting Genetic Algorithm II (NSGA2) algorithms NSGA2-CH and NSGA2-CHS were first introduced in [1] and further validated in [2]. In this work we analogously modified NSGA3 with the same capabilities of NSGA2-CHS obtaining NSGA3-CHS. For this purpose we designed a more general algorithm: NSGA*.

Supplementary Pseudocode 1 NSGA* class definition.

```
class Nsga* inherits MultiObjectiveOptimizer

   method new(
         nGenerations, popsize, featureImportance,
         sort, tournament, mutation,
         cloneRepurposing):
      self.nGenerations = nGenerations
      self.popsize = popsize
      self.featureImportance = featureImportance
      self.sort = sort
      self.tournament = tournament
      self.mutation = mutation
      self.cloneRepurposing = cloneRepurposing

   method optimize(objectives, trainingData):
      hof = createNewHallOfFame()
      featImp = self.featureImportance(trainingData)
      pop = createNewRandomIndividuals(
            featImp, self.popsize)
      for 1::self.nGenerations:
         pop = evaluate(pop, objectives, trainingData)
         pop = self.sort(pop)
         offspring = self.tournament(pop)
         offspring = crossover(offspring)
         offspring = self.mutation(offspring)
         pop = concat(pop, offspring)
         if self.cloneRepurposing:
            pop = ReplaceClonesWithNewIndividuals(
                  pop, featImp)
         pop = evaluate(pop, objectives, trainingData)
         hof = updateHallOfFame(hof, pop)
         pop = self.sort(pop)
         pop = pop[1..self.popsize]
      return hof
```

Supplementary Pseudocode 1 describes the algorithm NSGA*, a further generalization of the generalized Nsga2 algorithm presented in [1]. NSGA* generalizes NSGA2 and NSGA3, together with their modifications NSGA2-CH, NSGA2-CHS, NSGA3-CH, and NSGA3-CHS. The differences between the algorithms derived from NSGA2 and NSGA3 reside in the tournament and in the sort routines. NSGA2 uses a binary tournament by rank in the sorted population [3]. NSGA3 uses a random tournament where pairs are selected randomly with replacement [4]. Both NSGA2 and NSGA3 use a hierarchical sort where individuals are first sorted by their domination front. The secondary sorting is different: it is based on the crowding distance in NSGA2 [3] and on reference point niching in NSGA3 [4]. The modifications CH and CHS use a primary sorting according to the clone index [1], and the original sorting of NSGA2 or NSGA3 as secondary sorting.

The class `Nsga*` inherits from `MultiObjectiveOptimizer`, so it can be used by itself or as wrapped algorithm inside DOSA-MO. `Nsga*` has a constructor that memorizes the algorithm-specific parameters and strategies: the number of generations, population size, feature importance function, sorting algorithm, tournament strategy, mutation operator, and flag for the use of clone repurposing. Since the DOSA-MO uses adjusted objective functions, NSGA* takes an `objectives` object in input. It is used by the `evaluate` function and represents the NSGA* ability to run with different objective functions. The algorithm is similar to generalized NSGA2 [1], but with the added possibility of personalizing the tournament strategy, sorting algorithm and objectives. It can be used as inner MO optimizer by the DOSA-MO and can be specialized also as NSGA3, NSGA3-CH, and NSGA3-CHS.

1.2 Considered adjusting regression models

Five regression models have been considered in the experimental validation for adjusting the fitness functions. All of them use the sample weights, computed by the DOSA-MO as the partial derivatives of the HV as explained above.

Dummy. The simplest regression model learns the weighted median.

ptree. The pruned decision tree regression model, minimizing the weighted absolute error. The tree is pruned with the Minimal Cost-Complexity Pruning algorithm [5]. The complexity parameter for the pruning is optimized by running a 5-fold CV on its training data.

*https://orcid.org/0000-0003-4852-2310

†Corresponding Author: Vittorio Fortino, Institute of Biomedicine, University of Eastern Finland, Kuopio, 70211, P.O. Box 1627, Yliopistonranta 1, e-mail: vittorio.fortino@uef.fi, https://orcid.org/0000-0001-8693-5285

RFReg. The random forest regression, minimizing the weighted absolute error.

SVR. The epsilon-support vector regression with Gaussian kernel type [6]. It uses an l2 regularization penalty. We use the default parameters of $C = 1$ and $\epsilon = 0.1$.

rSVR. The randomized SVR uses random search with 5-fold CV on its training data to optimize the parameters C and ϵ.

We include in the tests the unadjusted NSGA3-CHS, that is equivalent to use regression models that predict always 0 (shortened as "zero").

1.3 Limit the computational overhead for adjustments

Running the tuning optimizer in a nested k-fold CV in order to generate the samples used for training the regression models imposes a computational overhead with respect to the cost of running the main optimizer without any adjustment to the fitness functions. An high overhead could make the DOSA-MO impractical in real cases. In our experimental validation, that uses GAs as inner MO optimizers, we limited the computational overhead by using in the tuning optimizer a smaller population and less generations than in the main optimizer.

The simplifying assumption is made for the computational cost of the GAs to be proportional to the number of individual evaluations multiplied by the number of samples used for evaluating the individuals (in training algorithms the cost is typically at least linear in the number of samples, since the algorithm has to at least iterate through them), and for the number of evaluations to be in turn proportional to the population size multiplied by the number of generations (this is in fact an upper bound considering that individuals equal to previously evaluated ones might not need to be evaluated again). We define a parameter μ as the desired ratio between the computational cost of the tuning phase with respect to the main optimization phase.

In order to have a computation time of the DOSA-MO approximately double than the time required by the unadjusted optimizer, μ is set to 1 in our experiments.

In the case of internal k-fold CV, let η be the number of external folds. We use the same number of folds also for the generation of the training samples for the regression models, so the tuning optimizer is executed that number of times for each execution of the DOSA-MO. Let ρ be the population size for the main optimizer, and ρ' the population size for the tuning optimizer. We compute ρ' with the following equation.

$$\rho' = \left\lfloor \rho \sqrt{\frac{\mu}{\eta - 1}} \right\rceil \tag{1}$$

Where $\lfloor \cdot \rceil$ is the round to the nearest integer operation.

Let γ be the number of generations for the main optimizer, and γ' the number of generations for the tuning optimizer. Similarly, we compute γ' with the following equation.

$$\gamma' = \left\lfloor \gamma \sqrt{\frac{\mu}{\eta - 1}} \right\rceil \tag{2}$$

According to the previous assumptions, the cost of running the main optimizer c is $c = \rho\gamma m$, with m being the number of samples. The cost of running all the iterations of the tuning optimizer c' is the following.

$$c' = \eta\rho'\gamma' \frac{m(\eta - 1)}{\eta} = \left\lfloor \rho \sqrt{\frac{\mu}{\eta - 1}} \right\rceil \left\lfloor \gamma \sqrt{\frac{\mu}{\eta - 1}} \right\rceil m(\eta - 1) \tag{3}$$

Ignoring the round operations that have just a small contribution it is easy to verify the desired ratio.

$$c' \approx \rho\gamma\mu m = \mu c \tag{4}$$

So the computational cost of using the adjusted optimizer wrapper, including the main optimizer, is approximately equal to $(1 + \mu)c$, or even lower if the cost of evaluating individuals grows more than linearly in the number of samples.

The external validation is faster since it does not have an outer k-fold CV. For the external validations, we have arbitrarily set $\eta = 5$, resulting in samples for regression training to be acquired from 5 folds.

1.4 Computing the fitness functions variance

One of the meta-features of the solutions, used for prediction by the adjustment regression models, is the SD of the original fitness.

Collecting the fitnesses of an individual (feature set in our case study) on the different folds and computing the SD of a performance metric (for example, the balanced accuracy or the c-index), would have a limited precision because of the number of the folds, that is necessarily contained to have an acceptable computational time. On the other hand, increasing the fold count would heighten computational demands, as the inner models need to be trained for each fold. Additionally, each fold fitnesses would be estimated on a smaller left-out sample set. Moreover, performing repeated CV would increase the number of evaluations but the same test samples would be reused.

Our method to calculate each solution's fitness variances addresses these issues by performing bootstrap analysis within each fold of the k-fold CV, followed by aggregation of the results across all folds. The following steps describe how one of the fitnesses for one of the individuals is computed.

1. For each of the folds there are training and testing samples. The inner model is optimized on the training data. The fitness is computed on the whole testing data, then by using bootstrap on the testing data, the variance for the fitness is computed.

2. The results from each of the folds are aggregated considering the folds as strata in a stratified bootstrap. The variances in the different folds are assumed uncorrelated and combined with the equation for the variance of the mean of uncorrelated random variables: the variance of the mean is the sum of the variances divided by the square of the number of folds.

When cross-validating, there are two sources of performance variance: the composition of the training set affects the training process thus can lead to different predictors, and indirectly to different expected performance, while the composition of the test set directly impacts the expected performance [7]. It is known that there is no unbiased estimator of the variance of k-fold CV [8]. The described procedure accounts for the variance explained by the limited number of test samples, but only partly for the variance explained by the limited number of training samples, since the training samples are reused in different folds (this is a well known unavoidable limitation in k-fold CV [9]) and the bootstrap is applied on the test sets but not on the training sets, to avoid incurring in infeasible computational costs. Despite the limitations, this estimation of the fitness variance appeared predictive of the overestimation in our experiments, thus justifying its inclusion in the meta-features for the overestimation prediction.

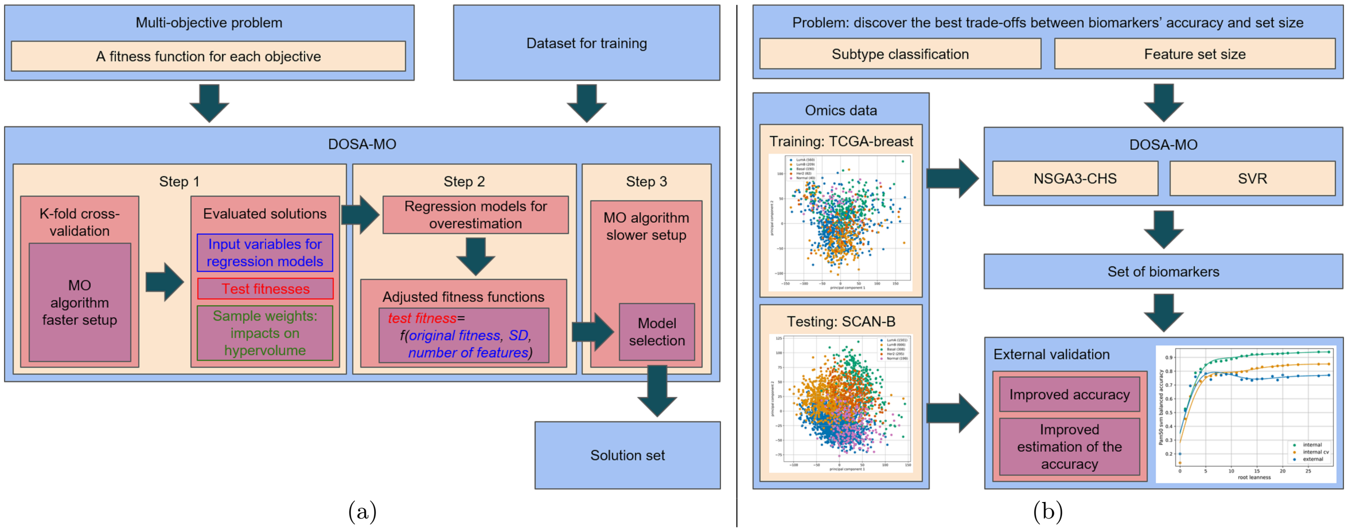

Figure S1: Depiction of DOSA-MO architecture and a use case for external validation of breast cancer biomarkers. (a) Sequence of operations of DOSA-MO. A MO problem is defined with multiple objectives. Each of them has its own fitness function. A dataset is fed to DOSA-MO for its optimization process in 3 steps. In step 1 it performs a k-fold CV with a wrapped MO optimizer and collects the solutions from all the folds. From each solution and objective a sample is constructed. It has the fitness expected by the wrapped MO, its SD, and the feature set size as independent variables, the overestimation (expected fitness minus fitness assessed on left-out set) as dependent variable, and the partial derivative on the HV with respect to this fitness measurement as sample weight. In step 2 these samples are used to train regression models for overestimation, and new adjusted objective functions are created. In step 3 a wrapped MO optimizer is run with the adjusted objective functions, impacting how the models are selected. DOSA-MO outputs a set of solutions. It might be beneficial to use a faster wrapped optimizer in step 1 than in step 3 since step 1 uses k-fold CV. (b) Use case for external validation of breast cancer biomarkers identified by DOSA-MO. A MO problem is defined with 2 objectives: cancer subtype classification and parsimony in the feature set size. TCGA breast omics data is fed to DOSA-MO for its optimization process. In this example it uses the genetic algorithm NSGA3-CHS as wrapped MO optimizer and support vector regressions to adjust the objective functions. The output of DOSA-MO is a set of biomarkers, each one with its own set of genes and expected accuracy. The SCAN-B dataset is used to externally validate the set of solutions. Both the accuracy and its estimation improve with respect to the biomarkers identified by NSGA3-CHS alone (Section 3).

1.5 Architecture and use case

Fig. S1 (a) shows the steps of the DOSA-MO algorithm, while the flow of a DOSA-MO use case is depicted in Fig. S1 (b).

1.6 Benchmarking datasets: description and pre-processing

We conducted benchmark studies with two primary goals: firstly, to identify gene expression-based biomarker sets for classifying cancer subtypes, and secondly, to determine similar biomarker sets for survival prediction in kidney and breast cancer patients.

Both the kidney and breast cancer case studies were addressed using k-fold CV on TCGA [10] data. Additionally, the breast cancer case study includes an external validation with training on TCGA data and testing on SCAN-B data [11]. The latter test is particularly important as it demonstrates that the proposed algorithm can also be utilized by training predictive models for both classification and overestimation within a specific cohort (e.g. TCGA) and applying them in external cohorts (e.g. SCAN-B).

TCGA transcriptomic datasets were downloaded with the curatedTCGAData R-package version 2.0.1 from assays of type RNASeq2GeneNorm [12]. The retrieved data consists of upper-quartile-normalized TPM values. They were log-transformed by applying $log_2(x+1)$.

The external gene expression-based transcriptomic dataset for breast cancer was obtained from the Gene Expression Omnibus (GEO) database (GSE96058) collected from the SCAN-B consortium. It includes FPKM log-transformed gene expression profiles. This data was already log-transformed, and we did not apply our own log-transformation to it. We applied for each value x the function $max(x, 0)$, then we excluded the genes with less than 30% non-zero values.

For our study, we utilized TCGA datasets specific to cancer types. For the breast cancer case study, we used the TCGA-BRCA dataset. Additionally, for kidney cancer, we considered a compendium of TCGA datasets, which includes: KICH (Kidney Chromophobe), KIRC (Kidney Renal Clear Cell Carcinoma), and KIRP (Kidney Renal Papillary Cell Carcinoma). Furthermore, our analysis for kidney cancer focused on identifying genes with a high likelihood of validation through PCR or immunohistochemistry laboratory tests. To this end, we utilized the 'pathology.tsv' file from The Human Protein Atlas which lists proteins detectable in human tumor tissues. To quantify overall detection levels, we summed the counts from 'High', 'Medium', and 'Low' detection categories for each sample, setting 3 as the minimum threshold for total detection level.

Each transcriptomic dataset was filtered by removing genes with zero variance and gene expression values were standardized before use. For both TCGA and SCAN-B, cancer patient samples were categorized based on the PAM50 cancer subtype signature, which is used to determine specific molecular subtypes of breast cancer. The subtypes identified by PAM50 that were used for our experiments are: Luminal A (LumA), Luminal B (LumB), HER2-enriched (Her2), Basal-like (Basal, which is often referred to as triple-negative) and normal-like (Normal) cancer. For the task of classifying cancer subtypes in TCGA kidney cancer patients, we focused on distinguishing clear-cell renal cell carcinoma (ccRCC), chromophobe renal cell carcinoma (ChRCC), and papillary renal cell carcinoma, which was further divided into two subtypes based on recent studies identifying distinct clinical categories. These are referred to as PRCC T1 and PRCC T2. Additionally, we included samples of normal non-cancerous tissues. This classification system is based on a study published by Ricketts et al. [13]. Overall survival data for TCGA kidney cancer is from Liu et al. [14].

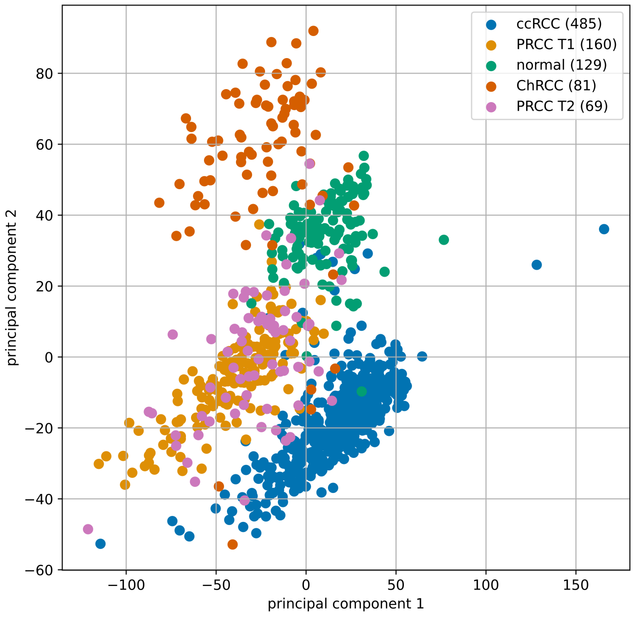

Figure S2: First two principal components with subtypes of all the TCGA kidney samples.

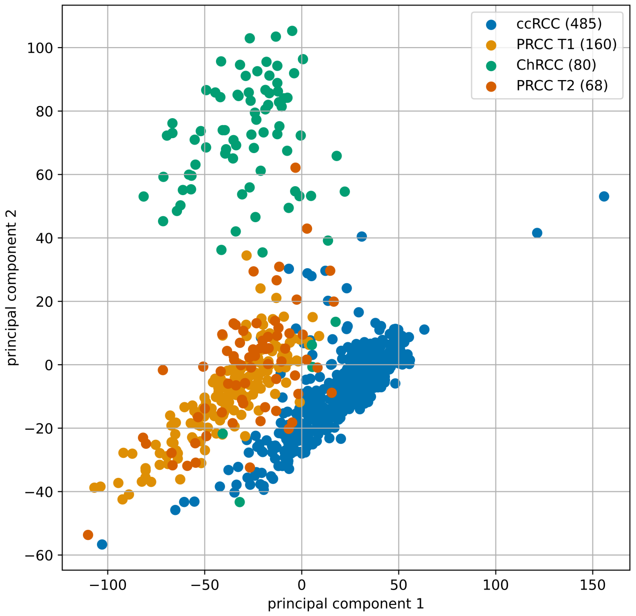

Figure S3: First two principal components with subtypes of the TCGA kidney samples with survival labels.

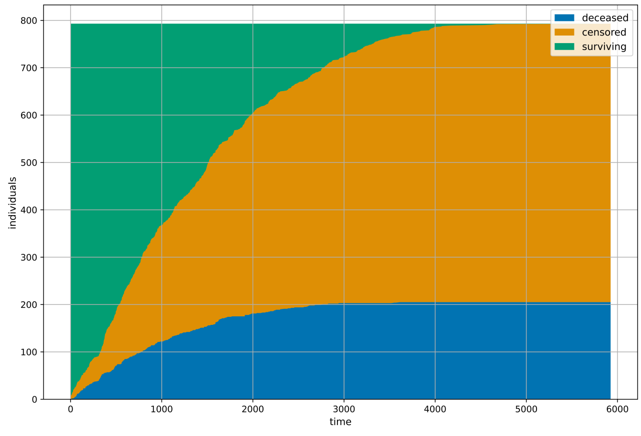

Figure S4: TCGA kidney survival outcomes.

The TCGA kidney dataset at the end of curation includes 5 classes and a total of 924 samples. Supplementary Fig. S2 shows the first two principal components (after standardization) and the number of mRNA-based profiles for each kidney cancer subtype. When the prediction of overall survival was included in the objectives, a smaller dataset of 793 samples was used in order to avoid the unlabelled samples (Supplementary Fig. S3). This led to the removal of all the samples of normal (healthy) tissue, because in TCGA they are collected from healthy areas of cancer patients. Additionally, two samples are excluded because they are missing the survival information. Supplementary Fig. S4 shows the distribution of the survival outcomes in time.

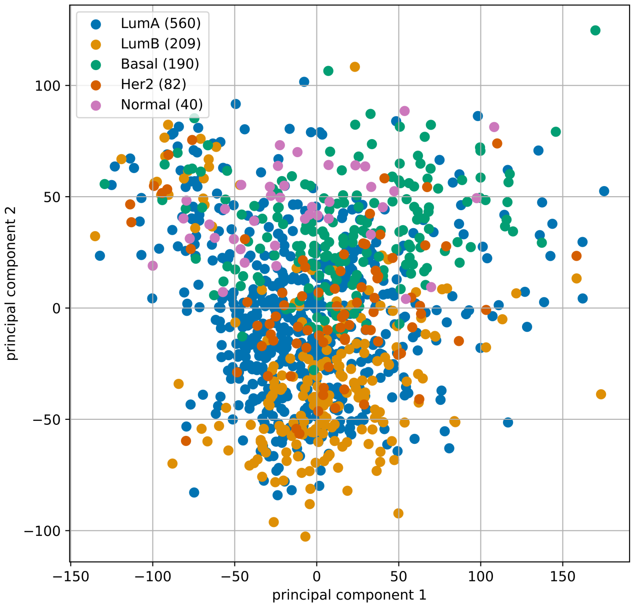

Figure S5: First two principal components with subtypes of the TCGA breast samples.

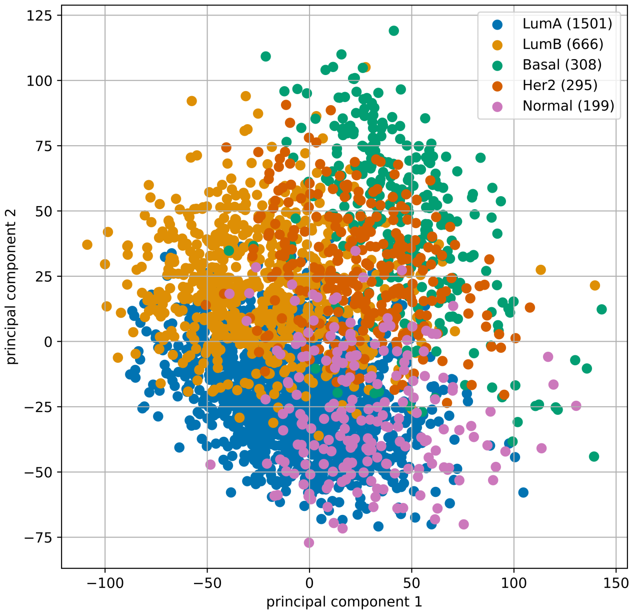

Figure S6: First two principal components with subtypes of the SCAN-B samples.

The TCGA breast dataset at the end of curation includes 5 classes and a total of 1081 samples. Supplementary Fig. S5 shows the first two principal components (after standardization) and the number of mRNA-based profiles for each breast cancer subtype in TCGA data. The SCAN-B dataset

at the end of curation includes 5 classes and a total of 2969 samples (Supplementary Fig. S6).

1.7 Experimental setup

Our tests include three datasets: TCGA kidney used for k-fold CV, TCGA breast used for k-fold CV and for the training part of external validation, and SCAN-B used for the testing part of external validation. The datasets and their preprocessing are described in Supplementary Section 1.6.

Our MO problems include three objectives that measure the ability to classify the cancer subtypes, the ability to predict the overall survival, and the feature set size. These objectives are measured in the $[0, 1]$ interval as per assumption of the algorithms. Subtype classification is measured with the balanced accuracy, survival prediction with the c-index, while the feature set size with the novel metric root-leanness. It was measured with leanness in a previous study [1]. The leanness is a value between 0 and 1 with higher values for solutions with less features. It is defined as $1/(n+1)$, with n being the number of features. The leanness decreases sharply as the feature set size increases; consequently, the CHV is strongly impacted by the accuracy of the smallest biomarkers that use 1 or 2 features. Taking this into account, Cattelani et al. [2] provided an additional evaluation of the same solution sets by using a different measure for the impact of the number of features: $1/(\sqrt{n}+1)$. This soft-leanness decreases less sharply as the set size increases. The root-leanness is even smoother than the soft-leanness and is defined as the root of the leanness: $\sqrt{1/(n+1)}$.

The program performs a 3-fold CV repeated 3 times for internal k-fold CV on the kidney dataset, while, for uniformity with previous works [1, 2], a 5-fold CV on the breast dataset. The MO optimizer used is the DOSA-MO (Section 2.2), wrapping NSGA3-CHS (Supplementary Section 1.1) as both tuning optimizer and main optimizer. The hyperparameters of NSGA3-CHS used as main algorithm are population 500, generations 500, and 3 folds used for the evaluation of the individuals. The hyperparameters related to the tuning phase of the DOSA-MO are set as explained in Supplementary Section 1.3. Each test is repeated 6 times, one for each of the 5 regressors described in Supplementary Section 1.2, and one for the zero regressor (equivalent to the unadjusted NSGA3-CHS).

In this study, for the task of cancer subtype classification, NB and SVM were selected as the inner models. Meanwhile, Cox was employed as the inner model for the task of cancer survival prediction. The SVM uses the Radial basis function kernel, balanced class weight, and l2 regularization parameter $C = 1$.

The considered experimental setups are listed in Table 1.

In each k-fold CV execution, samples are stratified based on the included objectives. For balanced accuracy, stratification is by cancer subtype, and for overall survival, by survival status and time (binned into high or low for evenly distributed death events). If both these objectives are present, stratification combines both criteria.

During each GA call, for classification objectives, features not passing an ANOVA test (p-value 0.05) are removed from the current training samples. For survival objectives, features that fail a Wald test (p-value 0.05) in a Cox regression are discarded. If both objectives apply, features failing both tests are dropped.

For each combination of validation type, datasets, objectives, adjusting regression model, and classification inner model, we calculated the MOPE and P_{Δ} to assess the difference between expected and actual performance on the test samples. The CHV was also computed as an overall performance indicator for the solution sets. To the best of our knowledge, it is the only proposed generalization of both HV to CV scenarios and of single-objective CV to MO [2]. CHV takes into account the differences between the performance expected by the optimization algorithm and measured on the test samples and preserves the HV appreciated properties, in particular the strict monotonicity [15]: if a set of solutions is strictly better, the CHV is guaranteed to be higher.

2 Supplementary results

2.1 Meta-features correlation

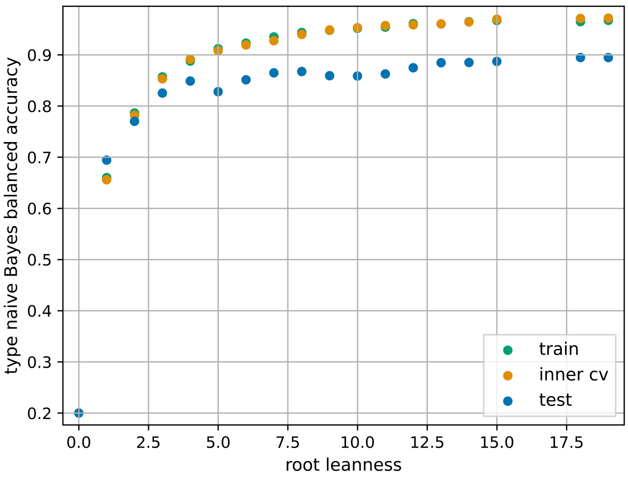

Figure S7: Example of scatter plot depicting solutions from CV on kidney cancer transcriptomics data. MO optimization of balanced accuracy for subtypes prediction and root-leanness. Horizontally, the number of features is shown for simplicity. For each solution it is shown the performance measured in the inner CV, i.e. the performance expected by the optimizer, the performance of the model trained on the whole training set and tested on the same set, and the performance of the same model on the testing set. The number of features, the expected performance and the overestimation are correlated.

Supplementary Fig. S7 shows an example of solution set performance after executing optimization with NSGA3-CHS for kidney cancer subtypes biomarker discovery, without any performance adjustment. The optimizer explored the best trade-offs between balanced accuracy and feature set size, and used 3-fold CV internally to evaluate the solutions. The overestimation (difference between the yellow and blue dots) of the balanced accuracy increases as the number of features increases. Analogously, the overestimation increases when the estimation increases. Also the variance of the estimation, computed by bootstrapping the samples, is correlated with the overestimation: the 9 p-values of the Kendall correlation for a 3-fold CV repeated 3 times are 0.008, 0.000, 0.068, 0.020, 0.006, 0.012, 0.003, 0.034, and 0.021.

2.2 Effect of DOSA-MO on solution sets in external validation

In this section, we examine a case study that utilizes two distinct cohorts, TCGA breast and SCAN-B, for external validation in biomarker discovery. The approach focuses on learning models and overestimation during biomarker identification within one cohort (like TCGA) and then applying this knowledge to another cohort (such as SCAN-B). This shows the reliability of biomarker discovery across different cohorts.

As previously shown, in the external validation for classification of breast cancer subtypes the DOSA-MO with SVR as fitness adjustment regression model and SVM as inner classifier provides the best overall performance measured with

the CHV, while, keeping the SVM as inner classifier, using the RFReg it is possible to obtain the more accurate prediction of the performance on a new cohort, with respect to both MOPE and P_{Δ}. The effect of the adjustment can be appreciated by looking at the solution sets presented in Fig. 2. The balanced accuracy expected by the optimizer ("internal cv" in the figures) is monotonically increasing with the number of features because the solutions returned by the algorithm are non-dominated. The estimation error, i.e. the vertical distance between the balanced accuracy expected by the optimizer using only the training samples and the balanced accuracy measured on the external dataset, is lower when using the DOSA-MO.

2.3 Identified biomarkers

For the problem of breast cancer subtype classification, running the optimization algorithms on TCGA data and testing the results on SCAN-B data has led to the identification of externally validated biomarkers that were not reported in previous studies. The performance of the biomarkers with the best trade-offs between feature set size and balanced accuracy, as measured on the external dataset SCAN-B, is shown for each combination of inner model and adjuster regression model in Supplementary Fig. S8. Consistently with the CHV results shown in Fig. 5 (f), the combination of SVM and SVR can be considered as the best one providing fast improving performances from 1 to 4 genes (0.79 balanced accuracy with 4 genes). Other algorithms take the lead when more genes are considered but the growth in balanced accuracy is comparatively slow.

Table S1 shows the best performing biomarkers, as measured on the external dataset SCAN-B. All selected biomarker models include markers, such as ESR1, FOXC1 and MIA that are consistent with findings from previous studies [16, 17, 2]. Among all the selected biomarkers, the one representing the most balanced trade-off is the biomarker set including the genes ESR1, FOXC1, KRT5, TPX2, which achieves 0.793 balanced accuracy with only 4 genes, considering that the maximum achieved balanced accuracy for the breast cancer-related gene sets is 0.807 on the external set. To the best of our knowledge, this is a new biomarker discovered by the DOSA-MO algorithm.

Supplementary Fig. S9 shows a detailed comparison between the performances in external validation of all the gene sets selected with or without DOSA-MO for each breast cancer subtype. The results shown are obtained with the SVM classifier and, when using DOSA-MO, the SVR regressor, the models that provided the best overall performance according to the CHV (Fig. 5 (f)). The reported all-vs-rest statistics include the balanced accuracy, the precision and the recall. Similar plots for all the considered datasets, objectives and algorithms are available in the project public repository (see Supplementary Section 3).

For each of the 5 experimental setups that are based on k-fold CV, the MO optimizers have also been run using the whole dataset as training set. For each experimental setup, the optimizer that achieved the highest CHV metric measured with k-fold CV (see Fig. 5) has been chosen, and the biomarkers that it selects, after running it on the whole dataset, including their gene sets and expected performance, are reported in the project public repository (see Supplementary Section 3).

Table S2 reports the best-performing biomarkers for the classification of kidney cancer subtypes identified by the best performing algorithm according to the CHV measured with repeated 3-fold CV: NSGA3-CHS with NB as inner model and SVR used for overestimation adjustment. It should be noted that, for the kidney cancer case, there is no external validation set available. Therefore, the accuracy reported is the one estimated by DOSA-MO after running on the entire TCGA dataset. Among these gene sets, we find that the biomarker model including the genes CACNA1H, GLB1L, HPCAL1, NECAB3, PDAP1, SQSTM1, STC2, VIPR2, represents the set that best balances the trade-off between accuracy and size. To the best of our knowledge, this gene combination has not been previously identified. Of particular note is STC2, a known prognostic marker in renal cell carcinoma [18]. STC2 is linked to cellular adaptation to hypoxia, which is highly relevant in ccRCC due to its dependence on the VHL pathway and hypoxia response mechanisms. This biomarker has shown potential for identifying RCC subtypes, as demonstrated in recent studies [19]. Another relevant gene is SQSTM1, which plays a role in autophagy and cellular stress response. This gene is often upregulated in cancer cells as they adapt to hypoxic and nutrient-poor environments. In general, autophagy-related genes have been shown to discriminate between renal cell carcinoma subtypes [20]. Additionally, there are novel genes such as NECAB3 and PDAP1 whose expression appears to be useful in distinguishing chRCC and pRCC subtypes, based on data from UALCAN: An update to the integrated cancer data analysis platform [21].

2.4 Hypervolumes computed on the test sets

For completeness, we report also the HV computed on the test or external data for each experimental setup (Supplementary Fig. S10). Differently from the CHV, the HV computed on the test samples implicitly assumes the presence of a decision maker that chooses which solution to use with knowledge of the performance on the test data. Naturally, this is not true in a real application. Because of this, the HV computed on the test samples is not a generalization of CV to MO situations [2].

3 Public repository

The original gene expression data used in this study is from public repositories (TCGA breast, TCGA kidney and SCAN-B). A description of the datasets included in our benchmark and the preprocessing that we have applied is in Supplementary Section 1.6. The preprocessed data, source code, and detailed numerical results are available in a public server (`github.com/UEFBiomedicalInformaticsLab/BIODAI/tree/main/DOSA_MO`). In the public repository, the directory `R` contains R scripts for preprocessing. They are not necessary to reproduce our results since we also provide the preprocessed data, but they are included for transparency. The directory `py` contains all the Python scripts of the project. With the exception of the initial preprocessing in R, everything else is in the Python programming language (version 2.9+). The file `requirements.txt` in the project root directory contains a list with the necessary Python packages and their suggested versions. The directory `work` contains configuration files that can be used to run the programs with the same settings that we used for this work, the preprocessed input files, the results for each program execution that we performed, and summary plots, tables, and other report files for each considered experimental setup in an aggregated form. Documentation about how to start the programs and access the results is in the file `readme.md` in the root directory.

Each dataset has a label that is recognized by the programs. We list below the directories containing the input and result data. For a more detailed description see the repository documentation.

- The `work/[dataset]/input` directory contains the preprocessed input data for the dataset.

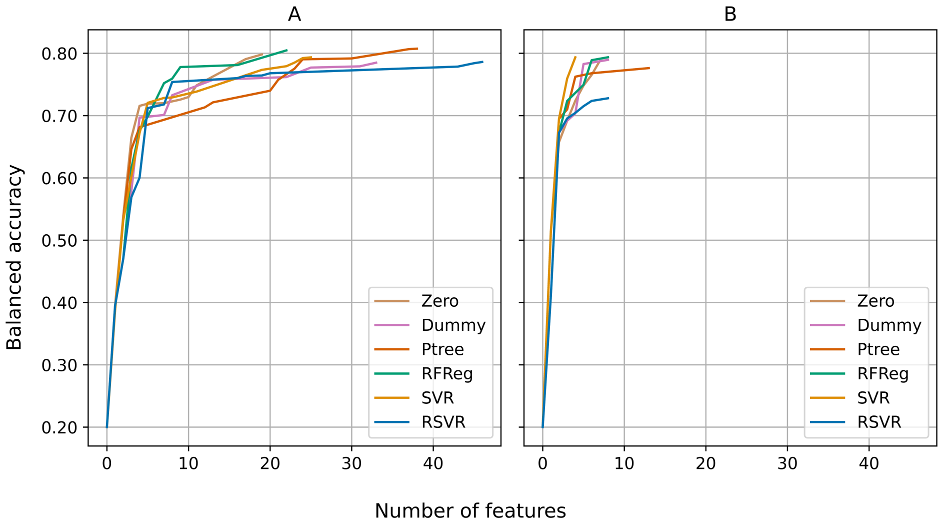

Figure S8: Performance of the best biomarkers for breast cancer subtype classification after optimizing on the TCGA dataset and testing on the SCAN-B dataset, with all combinations of inner model and adjuster regression model. The balanced accuracy is computed on the external dataset. (A) NB as inner model. (B) SVM as inner model.

Number of genes	Balanced accuracy	Balanced accuracy per class	Genes	Balanced accuracy without DOSA-MO
2	0.695	Basal: 0.929<br>Her2: 0.850<br>LumA: 0.677<br>LumB: 0.751<br>Normal: 0.774	ESR1, FOXC1	0.692
3	0.759	Basal: 0.891<br>Her2: 0.832<br>LumA: 0.806<br>LumB: 0.850<br>Normal: 0.850	ESR1, MIA, TPX2	0.724
4	0.793	Basal: 0.934<br>Her2: 0.831<br>LumA: 0.821<br>LumB: 0.864<br>Normal: 0.881	ESR1, FOXC1, KRT5, TPX2	0.724
8	0.794	Basal: 0.962<br>Her2: 0.886<br>LumA: 0.808<br>LumB: 0.871<br>Normal: 0.803	CCNB2, DNAJA2, ERBB2, FOXC1, HIVEP2, IGF1R, KRT5, RERG	0.788
19	0.798	Basal: 0.963<br>Her2: 0.873<br>LumA: 0.857<br>LumB: 0.871<br>Normal: 0.800	ALAD, CDC25B, CDC7, CKS1B, DEFB1, ENPP1, ERBB2, ERI2, FGD3, FOXC1, IGF1R, IGFALS, KRT14, KRT5, NUDT6, RNF39, SERPINF2, TCF19, YPEL2	0.798
22	0.805	Basal: 0.962<br>Her2: 0.905<br>LumA: 0.860<br>LumB: 0.861<br>Normal: 0.795	ACSM5, ATG4B, AURKA, CKS1B, COL17A1, ERBB2, FAM189A2, FOXC1, IGF1R, MKNK2, MPHOSPH6, NUDT6, OSR1, OTUD7B, PDE3A, RUNDC1, S100A2, SEMA4D, TAF5, TCF19, TP63, WDHD1	0.798
37	0.807	Basal: 0.963<br>Her2: 0.888<br>LumA: 0.853<br>LumB: 0.857<br>Normal: 0.823	CDC25B, CDC7, CDK11B, CEBPD, CKS1B, CXCL11, DEFB1, ENPP1, ENTPD2, ERBB2, FADS1, FNBP1, FOXC1, FSTL3, GPR160, IGFALS, KRT5, LMF1, LRFN3, MBD6, MBOAT1, MIA, MYO1G, NUDT12, NUDT6, RCCD1, RGS22, RNF39, RPL22L1, SCAMP3, SCYL3, SERPINA6, SKA1, STAC2, TCF19, VIPR1, ZNF763	0.798
38	0.807	Basal: 0.967<br>Her2: 0.882<br>LumA: 0.849<br>LumB: 0.858<br>Normal: 0.827	APLN, CDC25B, CDC7, CDK11B, CEBPD, CKS1B, DEFB1, ENPP1, ENTPD2, ERBB2, FADS1, FCGR1A, FOXC1, FSTL3, GPR160, HIGD1B, IGFALS, KRT5, LMF1, LRFN3, MBD6, MBOAT1, MIA, MYO1G, NUDT12, NUDT6, RCCD1, RGS22, RNF39, RPL22L1, SCAMP3, SERPINA6, SKA1, STAC2, TCF19, TOX4, VIPR1, ZNF763	0.798

Table S1: Best biomarkers with 2+ genes for breast cancer subtype classification after optimizing on the TCGA dataset and testing on the SCAN-B dataset. For each biomarker, it is reported: the number of included genes, the balanced accuracy, the balanced accuracies one-vs-rest, the list of genes that compose the biomarker, and the best balanced accuracy obtained by feature sets of equal or lower cardinality identified without DOSA-MO. All the balanced accuracies of this table are measured on the external test set.

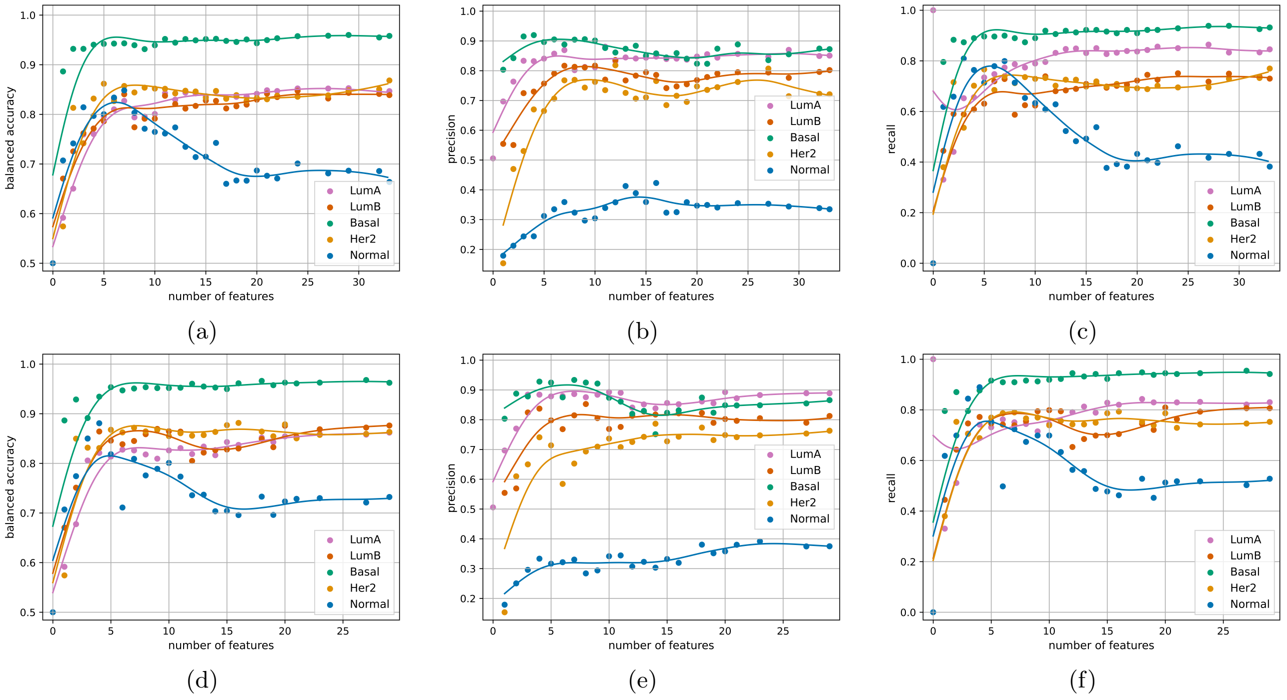

Figure S9: Balanced accuracy (a, d), precision (b, e) and recall (c, f) by class (one-vs-rest) for each solution, computed on SCAN-B data for breast cancer external validation of NSGA3-CHS using SVM as inner model, with DOSA-MO and SVR (d, e, f) or without DOSA-MO (a, b, c). The figures contain interpolating splines. (a) Balanced accuracy without DOSA-MO. (b) Precision without DOSA-MO. (c) Recall without DOSA-MO. (d) Balanced accuracy with DOSA-MO. (e) Precision with DOSA-MO. (f) Recall with DOSA-MO.

Number of genes	Expected balanced accuracy	Genes
2	0.698	ENPP3, TPK1
3	0.778	SPOCK2, STC2, TPK1
4	0.813	CCDC57, GLB1L, SQSTM1, STC2
5	0.822	ALDH9A1, NECAB3, RANBP3L, SQSTM1, STC2
6	0.835	CACNA1H, GLB1L, GLS, NECAB3, SQSTM1, STC2
7	0.845	CACNA1H, CAND2, GLB1L, HUNK, NECAB3, SQSTM1, STC2
8	0.850	CACNA1H, GLB1L, HPCAL1, NECAB3, PDAP1, SQSTM1, STC2, VIPR2
9	0.859	CAND2, GLB1L, HUNK, NECAB3, PGS1, PRTFDC1, SQSTM1, STC2, TRDMT1
10	0.863	CACNA1H, CAND2, GLB1L, HUNK, NECAB3, PDAP1, PNCK, PRTFDC1, SQSTM1, STC2
11	0.865	CACNA1H, CAND2, GLB1L, HUNK, NECAB3, PDAP1, PRTFDC1, SLC26A11, SQSTM1, STC2, VIPR2
12	0.869	AMT, CACNA1H, GLB1L, HPCAL1, HTATIP2, HUNK, NECAB3, NUDCD3, PRTFDC1, SQSTM1, STC2, VIPR2
13	0.871	ARMCX1, CACNA1H, GABARAPL1, GLB1L, HAX1, HUNK, NECAB3, PDAP1, PRTFDC1, SQSTM1, STC2, TRDMT1, VIPR2
14	0.874	AMT, CACNA1H, GABARAPL1, GLB1L, HAX1, HUNK, NECAB3, PRTFDC1, SLC26A11, SQSTM1, SRD5A1, STC2, TRDMT1, VIPR2
15	0.879	ARMCX1, CACNA1H, GABARAPL1, GLB1L, HAX1, HUNK, NECAB3, PDAP1, PRTFDC1, RPA2, SLC26A11, SQSTM1, STC2, TRDMT1, VIPR2
16	0.880	ARMCX1, CACNA1H, GABARAPL1, GDI2, GLB1L, HAX1, HUNK, NECAB3, PDAP1, PRTFDC1, RPA2, SLC26A11, SQSTM1, STC2, TRDMT1, VIPR2
17	0.881	ARMCX1, CACNA1H, FOS, GABARAPL1, GDI2, GLB1L, HAX1, HUNK, NECAB3, PDAP1, PRTFDC1, RPL21, SLC26A11, SQSTM1, STC2, TRDMT1, VIPR2
17	0.881	ARMCX1, CACNA1H, GABARAPL1, GDI2, GLB1L, HAX1, HUNK, NECAB3, PDAP1, PPM1G, PRTFDC1, RPA2, SLC26A11, SQSTM1, STC2, TRDMT1, VIPR2
18	0.881	ARMCX1, CACNA1H, GABARAPL1, GDI2, GLB1L, HAX1, HUNK, NECAB3, NRIP1, PDAP1, PPM1G, PRTFDC1, RPA2, SLC26A11, SQSTM1, STC2, TRDMT1, VIPR2
20	0.883	AMT, C1orf116, CACNA1H, EYA4, GABARAPL1, GLB1L, GLMN, GMEB1, HPCAL1, HUNK, MID1, NECAB3, PIR, PRTFDC1, SFRP1, SLC26A11, SQSTM1, SRD5A1, UBC, VIPR2
24	0.884	AMT, APOBEC3A, C1orf116, CACNA1H, CYB5B, EYA4, GABARAPL1, GLB1L, GLMN, HAX1, HPCAL1, HSPA12A, HUNK, MID1, NECAB3, PIR, PRTFDC1, PTMA, SFRP1, SLC26A11, SQSTM1, TTC27, UBC, VIPR2

Table S2: Best biomarkers with 2 to 24 genes for kidney cancer subtype classification after optimizing on the TCGA dataset with DOSA-MO wrapping NSGA3-CHS NB and using SVR for overestimation adjustment. For each biomarker, it is reported: the number of included genes, the expected balanced accuracy, and the list of genes that compose the biomarker. All the expected balanced accuracies of this table are measured during the optimization, with 3-fold CV followed by overestimation adjustment.

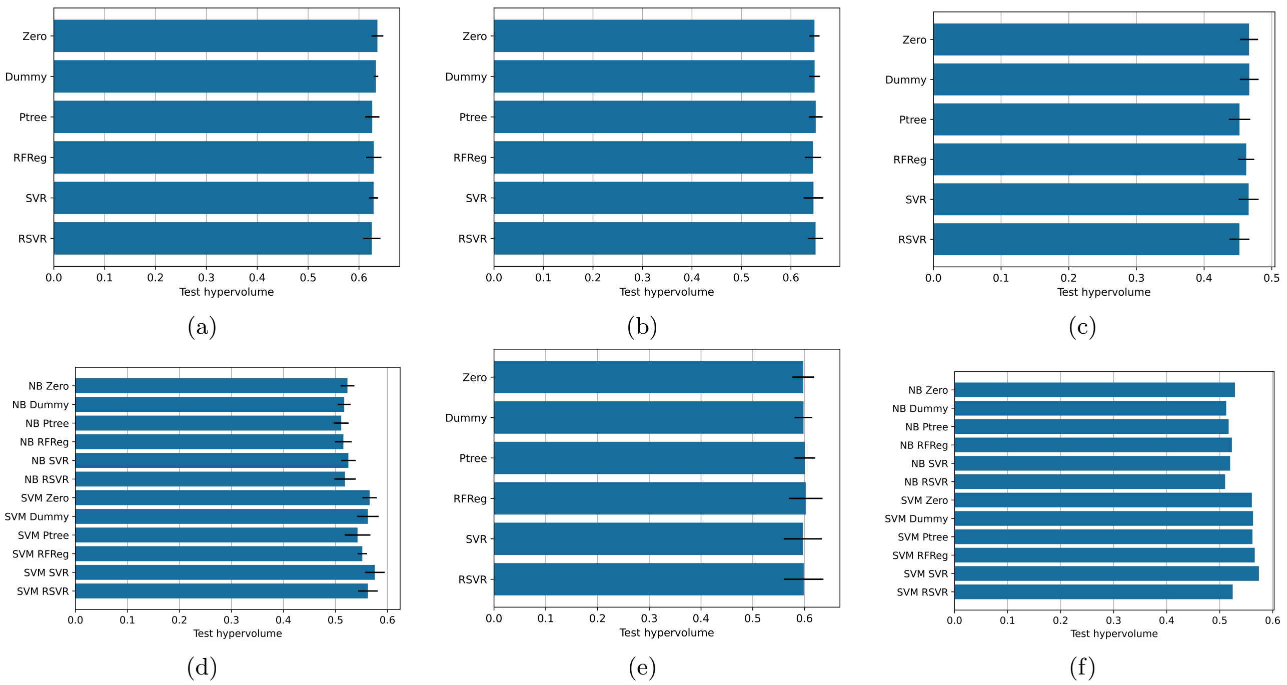

Figure S10: HV results computed on test folds for internal k-fold CV (a-e) and external data for external validation (f). (a) Kidney cancer, subtype classification and root-leanness. (b) Kidney cancer, overall survival prediction and root-leanness. (c) Kidney cancer, overall survival prediction, subtype classification and root-leanness. (d) Breast cancer, subtype classification and root-leanness. (e) Breast cancer, overall survival prediction and root-leanness. (f) External validation for breast cancer, subtype classification and root-leanness. Error bars represent SD between folds.

- The `work/setups/[dataset]` directory contains the configuration files for the runs that use the dataset for training. These are read by the programs to set their parameters.

- The `work/[dataset]/mrna` directory contains the detailed results of the runs that use the dataset for training. Each run has its own subdirectory.

The remaining directories contain aggregated plots and report files. See the repository documentation for a detailed list of the available contents. The repository has been tested for installation and execution by an external person without help using only the available documentation. The project is licensed under the terms of the MIT license.

4 Technical terms

The following is a list of the less common technical terms used in this paper. When they can have more than one possible meaning, we specify the one to which we refer to.

Abstract class. A high-level structure for a group of subclasses that share common methods or attributes, but the implementation of these methods may vary across subclasses.

ANOVA test. The ANOVA (Analysis of Variance) test is a statistical method used to compare the means of two or more groups of data sets to determine the extent of their difference.

Applicability domain. The characteristics of the training set on which the model has been developed, and for which it is applicable to make predictions for new data.

Balanced accuracy. A machine learning metric for binary and multi-class classification models, adjusted on imbalanced datasets by taking the average of sensitivity (true positive rate) and specificity (true negative rate).

Binary tournament. A binary tournament in genetic algorithms is a selection method where two individuals are randomly chosen from the population, and the one with the better fitness score is selected for the next generation.

Binned. A process that transforms continuous numerical variables into discrete categorical “bins”.

Biomarker. A measurable characteristic that reflects the physiological or pathological state of an organism, and these characteristics can be used to diagnose diseases, monitor treatment outcomes, or predict disease progression [22].

Biomarker discovery. The process of identifying and validating new biomarkers, often using machine learning techniques to analyse high-dimensional biological data [23].

Biomarker model. A model that uses biomarker data to make predictions.

Bootstrap. A statistical method used in machine learning for estimating the skill of machine learning models by resampling a dataset with replacement.

Cancer subtypes. The categorization of specific cancers into distinct groups based on molecular or other characteristics.

Concordance index. A measure of the predictive accuracy of a survival model, representing the probability that, for a randomly selected pair of individuals, the one who experiences the event of interest first has a higher risk score.

Cross hypervolume. Overall performance indicator for evaluating the solution set produced by a multi-objective optimizer using cross-validation. It generalizes both the hypervolume to cross-validation scenarios and single-objective cross-validation to multi-objective

problems [2]. It preserves the hypervolume appreciated properties, in particular the strict monotonicity [15]: if a set of solutions is strictly better, the cross hypervolume is guaranteed to be higher.

Cross-validation. A technique where a dataset is divided into two sections, one used to train a model and the other used to validate the model's performance.

Decision maker. In the context of multi-objective problems, refers to the entity (which could be a person, a group of people, or an algorithm) that, once the set of potential solutions is identified, often represented by a Pareto front, selects the most suitable solution based on its preferences [24, 25].

Estimate. A measure that quantifies how well a model is expected to perform on unseen data, often determined through techniques like cross-validation or using a separate test dataset.

Estimation error. The difference between the estimated performance of a model (based on training or validation data) and the actual performance of the model when applied to new, unseen data.

Evaluation. The process of assessing the performance of a model using certain metrics, which helps to understand how well the model generalizes to unseen data.

Expected performance. The anticipated accuracy or effectiveness of a model in making predictions on new, unseen data.

External set. A dataset that is different from the one used to train models, not just a different part of the same dataset.

External validation. The process of testing a model's performance using a dataset that was not used in the training process (not just a different part of the same dataset), providing a more realistic assessment of how the model will perform on new, unseen data [26].

Family of functions. Functions with a common definition that is parametric. The parameters can be other functions, like in the case of the CHV [2].

Feature selection. The process of selecting a subset of relevant features (variables, predictors).

Fitness estimation. The evaluation of a model's performance or the suitability of a solution in an optimization problem.

FPKM. FPKM (Fragments Per Kilobase Million) is a normalization method for quantifying gene expression from RNA-seq data, taking into account the effects of both sequencing depth and gene length.

Generations. In genetic algorithms, a generation refers to one iteration of the algorithm, where a population of solutions is evolved towards better solutions.

Genetic algorithm. An algorithm inspired by the process of natural selection, used to generate high-quality solutions to optimization and search problems by relying on biologically inspired operators such as mutation, crossover, and selection [27].

Hierarchical sort. A sorting process where data is organized in a hierarchical manner, meaning it's arranged according to levels of importance or significance.

Hyperparameter. A parameter that is set before the learning process begins and can directly affect how well a model trains. The set of features that are used is also a hyperparameter.

Hyperparameter configuration. The specific set of hyperparameters used in a machine learning model.

Hyperparameter tuning. The process of selecting the optimal values for a machine learning model's hyperparameters.

Hypervolume. In multi-objective optimization, the hypervolume indicator maps a solution set to the measure of the region dominated by that set [28].

Individual. In the context of genetic algorithms, an individual refers to a candidate solution to an optimization problem, which is analogous to a chromosome [27].

Inner model. A model to which a wrapper algorithm delegates the training and prediction for the considered hyperparameter configurations, which includes the feature sets.

K-fold cross-validation. A process where a dataset is randomly partitioned into k equal sized subsets, and a model or optimizer is trained on $k-1$ subsets and validated on the left-out subset, with this process repeated k times to ensure each subset is used for validation exactly once.

Left-out samples/set. Data points that are left out of the training set and used as the test set in cross-validation.

mRNA. Messenger ribonucleic acid.

Meta-feature. A property or characteristic that can be used to guide the algorithm selection or hyperparameter tuning process.

Model selection. The process of choosing one model from a set of candidate models based on their performance on a given dataset.

Molecular biomarker. A specific molecule or set of molecules used as features in a model to help with the diagnosis or prognosis of diseases or disorders [29, 23].

Molecular features. The specific characteristics or properties of molecules that are used as input data for models.

Molecular subtype. Subtype (of a disease) defined on the basis of molecular characteristics.

Moving target. A problem where the objective or the data changes over time, requiring the model to adapt.

Multi-objective feature selection. A process that aims to select the optimal subset of features considering multiple objectives, often involving trade-offs between conflicting objectives [30].

Multi-objective problem. A type of problem that involves optimizing multiple conflicting objectives simultaneously, returning a set of solutions that approximates as much as possible the Pareto front.

Mutation operator. In genetic algorithms, a mutation operator is used to introduce randomness and maintain diversity in the population by making small random changes in the individuals.

Non-dominated. A solution such that no other solution is equal or better with respect to all objectives, and strictly better with respect to a least one objective.

Non-dominated front. A set of solutions that are not dominated by any solution.

Objective. A specific goal that the models are designed to achieve, such as maximizing classification accuracy or survival prediction.

Objective function. A mathematical function used to quantify the performance of a solution with respect to an objective.

On-line learning. A learning method where data becomes available in a sequential order and is used to update the best predictor for future data at each step.

Optimizer. An algorithm that fine-tunes solutions with respect to the objectives of a problem.

Overall survival. In the context of survival analysis in machine learning, refers to the time until the occurrence of death [31].

Overestimation. A performance estimation that is higher than the real one.

Overfitting. Occurs when a machine learning model learns the detail and noise in the training data to the extent that it negatively impacts the performance of the model on new data.

Partial derivative. A partial derivative of a function of several variables is its derivative with respect to one of those variables, with the others held constant.

Pareto front. The set of solutions such that any other possible solution is dominated by them.

Partitioned. Divided into subsets so that the subsets are disjunct and contain all the elements.

Polymorphic. Polymorphism in programming refers to the ability of a variable, function, or object to take on multiple forms.

Population. In the context of GAs, the collection of individuals that are currently considered by the algorithm.

Primary sorting. In hierarchical sorting, the most important sorting criteria.

Random search. A strategy that uses random combinations of hyperparameters to identify the optimal answer.

Repeated CV. A technique where the CV process is repeated multiple times, providing a more robust estimate of the performance of a machine learning model.

Secondary sorting. In hierarchical sorting, the second most important sorting criteria.

Selected model. The model that gets selected from a collection of candidate models.

Single-objective problem. A problem where the goal is to optimize a single objective.

Solution. In the context of optimization algorithms, a possible solution to the optimization problem. It may be part of the final solution set, or a candidate solution during the execution of the algorithm.

Stratified bootstrap. A resampling technique where the dataset is divided into strata and samples are drawn independently from each stratum. The number of samples from each strata is relative to the strata prevalence.

Survival analysis. A subfield of statistics used in machine learning to analyze and model time-to-event data, dealing with censoring [31].

Survival status. In survival analysis, whether an event of interest (such as death) has occurred [31].

Test performance. The evaluation on a test set of a machine learning model's ability to make accurate predictions when presented with unseen data.

Tournament. In the context of GAs, a tournament involves running several "tournaments" among a few individuals (or "chromosomes") chosen at random from the population.

TPM. TPM (Transcripts Per Kilobase Million) is a normalized measure of gene expression that first normalizes for gene length and then for sequencing depth, making it easier to compare the proportion of reads that mapped to a gene in each sample.

Train performance. The evaluation of a machine learning model's ability to learn and make accurate predictions on the training dataset.

Training, validation, and test paradigm. A process where the dataset is divided into three subsets: training set (used to train the models), validation set (used to select the models), and test set (used to evaluate the final models' performance).

Upper-quartile-normalized. A normalization method where the gene counts are divided by the upper quartile of counts different from zero in the computation of the normalization factors associated with their sample.

Wald test. A hypothesis test done on the parameters calculated by the Maximum Likelihood Estimate (MLE) to check if the value of the true input parameters has the same likelihood as the parameters calculated by MLE.

Wrapper algorithm. An optimization algorithm that considers the hyperparameter tuning as a search problem, where different combinations are prepared, evaluated, and compared to other combinations.

References

[1] Luca Cattelani and Vittorio Fortino. Improved nsga-ii algorithms for multi-objective biomarker discovery. *Bioinformatics*, 38(Supplement_2):ii20–ii26, 09 2022. ISSN 1367-4803. doi:10.1093/bioinformatics/btac463. URL https://doi.org/10.1093/bioinformatics/btac463.

[2] Luca Cattelani, Arindam Ghosh, Teemu Rintala, and Vittorio Fortino. A comprehensive evaluation framework for benchmarking multi-objective feature selection in omics-based biomarker discovery. *IEEE/ACM Transactions on Computational Biology and Bioinformatics*, 21(6):2432–2446, 2024. doi:10.1109/TCBB.2024.3480150.

[3] K. Deb, A. Pratap, S. Agarwal, and T. Meyarivan. A fast and elitist multiobjective genetic algorithm: Nsga-ii. *IEEE Transactions on Evolutionary Computation*, 6(2):182–197, 2002. doi:10.1109/4235.996017.

[4] Kalyanmoy Deb and Himanshu Jain. An evolutionary many-objective optimization algorithm using reference-point-based nondominated sorting approach, part i: Solving problems with box constraints. *IEEE Transactions on Evolutionary Computation*, 18(4):577–601, 2014. doi:10.1109/TEVC.2013.2281535.

[5] L Breiman, JH Friedman, R Olshen, and CJ Stone. Classification and regression trees. 1984.

[6] Harris Drucker, Christopher J. C. Burges, Linda Kaufman, Alex Smola, and Vladimir Vapnik. Support vector regression machines. In M.C. Mozer, M. Jordan, and T. Petsche, editors, *Advances in Neural Information Processing Systems*, volume 9. MIT Press, 1996.

[7] Juan D. Rodriguez, Aritz Perez, and Jose A. Lozano. Sensitivity analysis of k-fold cross validation in prediction error estimation. *IEEE Transactions on Pattern Analysis and Machine Intelligence*, 32(3):569–575, 2010. doi:10.1109/TPAMI.2009.187.

[8] Yoshua Bengio and Yves Grandvalet. Bias in estimating the variance of k-fold cross-validation. In *Statistical modeling and analysis for complex data problems*, pages 75–95. Springer, 2005.

[9] Sylvain Arlot and Alain Celisse. A survey of cross-validation procedures for model selection. *Statistics Surveys*, 4(none): 40 – 79, 2010. doi:10.1214/09-SS054. URL `https://doi.org/10.1214/09-SS054`.

[10] Carolyn Hutter and Jean Claude Zenklusen. The cancer genome atlas: creating lasting value beyond its data. *Cell*, 173(2):283–285, 2018.

[11] Christian Brueffer, Johan Vallon-Christersson, Dorthe Grabau, Anna Ehinger, Jari Häkkinen, Cecilia Hegardt, Janne Malina, Yilun Chen, Pär-Ola Bendahl, Jonas Manjer, Martin Malmberg, Christer Larsson, Niklas Loman, Lisa Rydén, Åke Borg, and Lao H. Saal. Clinical value of rna sequencing–based classifiers for prediction of the five conventional breast cancer biomarkers: a report from the population-based multicenter sweden cancerome analysis network—breast initiative. *JCO precision oncology*, 2:1–18, Mar 2018. doi:10.1200/PO.17.00135. URL `https://doi.org/10.1200/PO.17.00135`. PMID: 32913985.

[12] Marcel Ramos, Ludwig Geistlinger, Sehyun Oh, Lucas Schiffer, Rimsha Azhar, Hanish Kodali, Ino de Bruijn, Jianjiong Gao, Vincent J Carey, Martin Morgan, et al. Multiomic integration of public oncology databases in bioconductor. *JCO Clinical Cancer Informatics*, 1:958–971, 2020.

[13] Christopher J Ricketts, Aguirre A De Cubas, Huihui Fan, Christof C Smith, Martin Lang, Ed Reznik, Reanne Bowlby, Ewan A Gibb, Rehan Akbani, Rameen Beroukhim, et al. The cancer genome atlas comprehensive molecular characterization of renal cell carcinoma. *Cell reports*, 23(1):313–326, 2018.

[14] Jianfang Liu, Tara Lichtenberg, Katherine A Hoadley, Laila M Poisson, Alexander J Lazar, Andrew D Cherniack, Albert J Kovatich, Christopher C Benz, Douglas A Levine, Adrian V Lee, et al. An integrated tcga pan-cancer clinical data resource to drive high-quality survival outcome analytics. *Cell*, 173(2):400–416, 2018.

[15] Miqing Li and Xin Yao. Quality evaluation of solution sets in multiobjective optimisation: A survey. *ACM Comput. Surv.*, 52(2), mar 2019. ISSN 0360-0300. doi:10.1145/3300148. URL `https://doi.org/10.1145/3300148`.

[16] Bingchen Han, Neil Bhowmick, Ying Qu, Stacey Chung, Armando E Giuliano, and Xiaojiang Cui. Foxc1: an emerging marker and therapeutic target for cancer. *Oncogene*, 36(28): 3957–3963, 2017.

[17] Yi Yu-Rice, Yanli Jin, Bingchen Han, Ying Qu, Jeffrey Johnson, Takaaki Watanabe, Long Cheng, Nan Deng, Hisashi Tanaka, Bowen Gao, et al. Foxc1 is involved in erα silencing by counteracting gata3 binding and is implicated in endocrine resistance. *Oncogene*, 35(41):5400–5411, 2016.

[18] Hellmuth-A. Meyer, Angelika Tölle, Monika Jung, Florian R. Fritzsche, Bernard Haendler, Ilka Kristiansen, Ariana Gaspert, Manfred Johannsen, Klaus Jung, and Glen Kristiansen. Identification of stanniocalcin 2 as prognostic marker in renal cell carcinoma. *European Urology*, 55(3):669–678, 2009. ISSN 0302-2838. doi:https://doi.org/10.1016/j.eururo.2008.04.001. URL `https://www.sciencedirect.com/science/article/pii/S0302283808004223`.

[19] Shuo Qie and Nianli Sang. Stanniocalcin 2 (stc2): a universal tumour biomarker and a potential therapeutical target. *Journal of Experimental & Clinical Cancer Research*, 41(1): 161, 2022.

[20] Zhaoyue He, He Liu, Holger Moch, and Hans-Uwe Simon. Machine learning with autophagy-related proteins for discriminating renal cell carcinoma subtypes. *Scientific reports*, 10(1):720, 2020.

[21] Darshan Shimoga Chandrashekar, Santhosh Kumar Karthikeyan, Praveen Kumar Korla, Henalben Patel, Ahmedur Rahman Shovon, Mohammad Athar, George J. Netto, Zhaohui S. Qin, Sidharth Kumar, Upender Manne, Chad J. Creighton, and Sooryanarayana Varambally. Ualcan: An update to the integrated cancer data analysis platform. *Neoplasia*, 25:18–27, 2022. ISSN 1476-5586. doi:https://doi.org/10.1016/j.neo.2022.01.001. URL `https://www.sciencedirect.com/science/article/pii/S147655862200001X`.

[22] Sandra Ng, Sara Masarone, David Watson, and Michael R Barnes. The benefits and pitfalls of machine learning for biomarker discovery. *Cell and Tissue Research*, 394(1):17–31, 2023.

[23] Ramon Diaz-Uriarte, Elisa Gómez de Lope, Rosalba Giugno, Holger Fröhlich, Petr V. Nazarov, Isabel A. Nepomuceno-Chamorro, Armin Rauschenberger, and Enrico Glaab. Ten quick tips for biomarker discovery and validation analyses using machine learning. *PLOS Computational Biology*, 18 (8):1–17, 08 2022. doi:10.1371/journal.pcbi.1010357. URL `https://doi.org/10.1371/journal.pcbi.1010357`.

[24] Diederik M. Roijers and Shimon Whiteson. *Multi-Objective Decision Problems*, pages 9–17. Springer International Publishing, Cham, 2017. ISBN 978-3-031-01576-2. doi:10.1007/978-3-031-01576-2_2. URL `https://doi.org/10.1007/978-3-031-01576-2_2`.

[25] Ignacy Kaliszewski, Janusz Miroforidis, and Dmitry Podkopaev. Multiple criteria decision making and multiobjective optimization - a toolbox. In Krassimir T. Atanassov, Janusz Kacprzyk, Andrzej Kałuszko, Maciej Krawczak, Jan Owsiński, Sotir Sotirov, Evdokia Sotirova, Eulalia Szmidt, and Sławomir Zadrożny, editors, *Uncertainty and Imprecision in Decision Making and Decision Support: Cross-Fertilization, New Models and Applications*, pages 135–142, Cham, 2018. Springer International Publishing. ISBN 978-3-319-65545-1.

[26] Chava L Ramspek, Kitty J Jager, Friedo W Dekker, Carmine Zoccali, and Merel van Diepen. External validation of prognostic models: what, why, how, when and where? *Clinical Kidney Journal*, 14(1):49–58, 11 2020. ISSN 2048-8505. doi:10.1093/ckj/sfaa188. URL `https://doi.org/10.1093/ckj/sfaa188`.

[27] Bushra Alhijawi and Arafat Awajan. Genetic algorithms: Theory, genetic operators, solutions, and applications. *Evolutionary Intelligence*, pages 1–12, 2023.

[28] Andreia P Guerreiro, Carlos M Fonseca, and Luís Paquete. The hypervolume indicator: Computational problems and algorithms. *ACM Computing Surveys (CSUR)*, 54(6):1–42, 2021.

[29] Chao Li, Zhenbo Gao, Benzhe Su, Guowang Xu, and Xiaohui Lin. Data analysis methods for defining biomarkers from omics data. *Analytical and Bioanalytical Chemistry*, 414(1): 235–250, 2022.

[30] Qasem Al-Tashi, Said Jadid Abdulkadir, Helmi Md Rais, Seyedali Mirjalili, and Hitham Alhussian. Approaches to multi-objective feature selection: A systematic literature review. *IEEE Access*, 8:125076–125096, 2020. doi:10.1109/ACCESS.2020.3007291.

[31] Ping Wang, Yan Li, and Chandan K Reddy. Machine learning for survival analysis: A survey. *ACM Computing Surveys (CSUR)*, 51(6):1–36, 2019.
